# Targeting autophagic cancer stem-cells to reverse chemoresistance in human triple negative breast cancer

**DOI:** 10.18632/oncotarget.16925

**Published:** 2017-04-07

**Authors:** Guilhem Bousquet, Morad El Bouchtaoui, Tan Sophie, Christophe Leboeuf, Cédric de Bazelaire, Philippe Ratajczak, Sylvie Giacchetti, Anne de Roquancourt, Philippe Bertheau, Laurence Verneuil, Jean-Paul Feugeas, Marc Espié, Anne Janin

**Affiliations:** ^1^ Université Paris Diderot, Sorbonne Paris Cité, Laboratoire Pathologie, Paris, France; ^2^ INSERM, Paris, France; ^3^ Université Paris 13, Villetaneuse, France; ^4^ AP, HP, Avicenne, Service Oncologie, Paris, France; ^5^ AP HP Hôpital Saint-Louis, Service Radiologie, Paris, France; ^6^ AP HP Hôpital Saint-Louis, Centre Maladies Sein, Paris, France; ^7^ AP HP Hôpital Saint-Louis, Service Pathologie, Paris, France; ^8^ INSERM, Paris, France

**Keywords:** breast cancer stem cells, TNBC, chemoresistance, autophagy, hypoxia

## Abstract

There is growing evidence for the role of cancer stem-cells in drug resistance, but with few *in situ* studies on human tumor samples to decipher the mechanisms by which they resist anticancer agents.

Triple negative breast cancer (TNBC) is the most severe sub-type of breast cancer, occurring in younger women and associated with poor prognosis even when treated at a localized stage.

We investigated here the relationship between complete pathological response after chemotherapy and breast cancer stem-cell characteristics in pre-treatment biopsies of 78 women with triple negative breast carcinoma (TNBC).

We found that chemoresistance was associated with large numbers of breast cancer stem-cells, and that these cancer stem-cells were neither proliferative nor apoptotic, but in an autophagic state related to hypoxia. Using relevant pharmacological models of patient-derived TNBC xenografts, we further investigated the role of autophagy in chemoresistance of breast cancer stem-cells. We demonstrated that hypoxia increased drug resistance of autophagic TNBC stem-cells, and showed that molecular or chemical inhibition of autophagic pathway was able to reverse chemoresistance.

Our results support breast cancer stem-cell evaluation in pre-treatment biopsies of TNBC patients, and the need for further research on autophagy inhibition to reverse resistance to chemotherapy.

## INTRODUCTION

According to cancer stem-cell theory, malignant tumors are heterogeneous, with a sub-population of tumor cells with stem features [1, 2]. There is growing evidence for the role of these sub-populations of cancer stem-cells in drug resistance [2, 3, 4 ], even though there are few *in situ* studies on human tumor samples. In human samples of renal cell carcinoma, we recently demonstrated that sunitinib, a tyrosine kinase inhibitor, was able to generate resistance to its own therapeutic effect in cancer stem cells *via* induced hypoxia [5]. In women with localized breast cancer, resistance to chemotherapy delivered before surgery is associated with larger numbers of cancer stem-cells after treatment [6].

The most severe breast cancer in younger women, associated with poor prognosis even when treated at a localized stage [7], is triple negative breast cancer (TNBC) defined by lack of expression of HER2, estrogen and progesterone receptors. The standard care for localized TNBC, when inflammatory or over 3 cm in diameter, is neoadjuvant chemotherapy before surgical removal of the primary tumor [8]. The absence of residual tumor at the time of surgery defines complete pathological response (pCR) [9], which is a relevant prognostic endpoint in clinical trials evaluating neoadjuvant chemotherapy for breast cancer [10]. The prognosis for women with pCR is excellent [9], but when pCR is not achieved, TNBC patients have a high relapse rate and poor survival [7]. Factors predicting pCR, and thus response to neoadjuvant chemotherapy, are still lacking.

The mechanisms by which cancer stem-cells resist anticancer agents are also not deciphered. Macro-autophagy, here referred to as autophagy, is a lysosomal pathway whereby a cell digests its own cytoplasmic components [11]. Initially described as a cell death mechanism [12], autophagy is also a cell survival pathway to escape programmed cell death and maintain cellular homeostasis, and that can be upregulated in quiescent cells [13]. It can thus be a survival process for cancer cells in response to intrinsic or extrinsic stress conditions, including hypoxic stress [14–16]. BNIP3L, an autophagy related protein, is linked to hypoxia: HIF1α induces its expression, leading to the activation of BECLIN1 and the autophagy pathway [16, 17]. Recent *in vitro* studies have also demonstrated the critical role of autophagy in the maintenance of breast cancer stem-cells [18, 19].

We investigated here the relationship between complete pathological response after neoadjuvant chemotherapy and breast cancer stem-cell characteristics in pre-treatment biopsies of 78 women with TNBC. Using patient-derived xenografts obtained from women with metastatic TNBC, we further investigated the role of autophagy in the chemoresistance of breast cancer stem-cells.

## RESULTS

### Patient follow-up, biopsies and pCR

Table 1 shows clinical data for 78 women with a ductal TNBC, prospectively enrolled in a registry and treated with neoadjuvant chemotherapy at Saint-Louis-Hospital between 2005 and 2011.

**Table 1:** Pretreatment characteristics and univariate associations with pCR

	pCR *n* = 20	non-pCR *n* = 58	*p*
**Clinical data**			
Median age (years)	48	52	ns
SBR grade			
I–II	30%	21%	ns
III	70%	79%	ns
Chemotherapy regimen			
SIM	25%	17%	ns
4 cycles EC - 4 cycles D	65%	69%	ns
Taxane-based	10%	14%	ns
Pathological data			
CD133-expressing cells	3.5% (± 0.9)	10.4% (± 4)	< 0.01
ALDH1-expressing cells	2.6% (± 2.8)	8.6% (± 5.9)	< 0.01
CD146-expressing cells	6.3% (± 3.8)	17.8% (± 6.3)	< 0.01
**CD133/ALDH1**			
co-expressing cells	1.2% (± 1.6)	6.3% (± 3.6)	< 0.01
**CD133/CD146**			
co-expressing cells	3.1% (± 1.1)	9.2% (± 3.1)	< 0.01
Necrosis			
Yes	25%	74%	< 0.01
No	75%	26%	< 0.01

SBR: Scarff, Bloom and Richardson grading system.

SIM: Dose-dense epirubicin-cyclophosphamide-based regimen (Lehmann-Che et al., 2010).

EC = epirubicin (100 mg/m<sup>2</sup>/cycle) and cyclophosphamide (500 mg/m<sup>2</sup>/cycle), one cycle every 21 days.

D = docetaxel (100 mg/m<sup>2</sup>/cycle), one cycle every 21 days.

The 78 pre-treatment biopsies, performed with a 16-gauge needle, provided samples with a mean length of 13.21 mm (± 1.56 mm), mean width 1.14 mm (± 0.12 mm), and mean surface area 15.05 mm<sup>2</sup> (± 1.88 mm<sup>2</sup>).

Breast surgery was performed after neoadjuvant chemotherapy for all patients. The same two pathologists (PB, AR) analyzed all surgical pieces. pCR, defined as absence of residual disease at the time of surgery in both breast and axillary lymph nodes, was found for 20 surgical pieces (25.6%). Over a median clinical follow-up of 33.2 months (range 4–75.5), the relapse rate was 14.8% for pCR patients, differing significantly (*p <* 0.01) from the 59.2% relapse rate for non-pCR patients (Supplementary Figure 1).

### Cancer stem-cell characterization and counts in patient tumor samples (Figure 1, Table 1)

We identified and counted breast cancer stem-cells in pre-treatment biopsies using CD133, CD146 and ALDH1 immunostaining.

**Figure 1:**
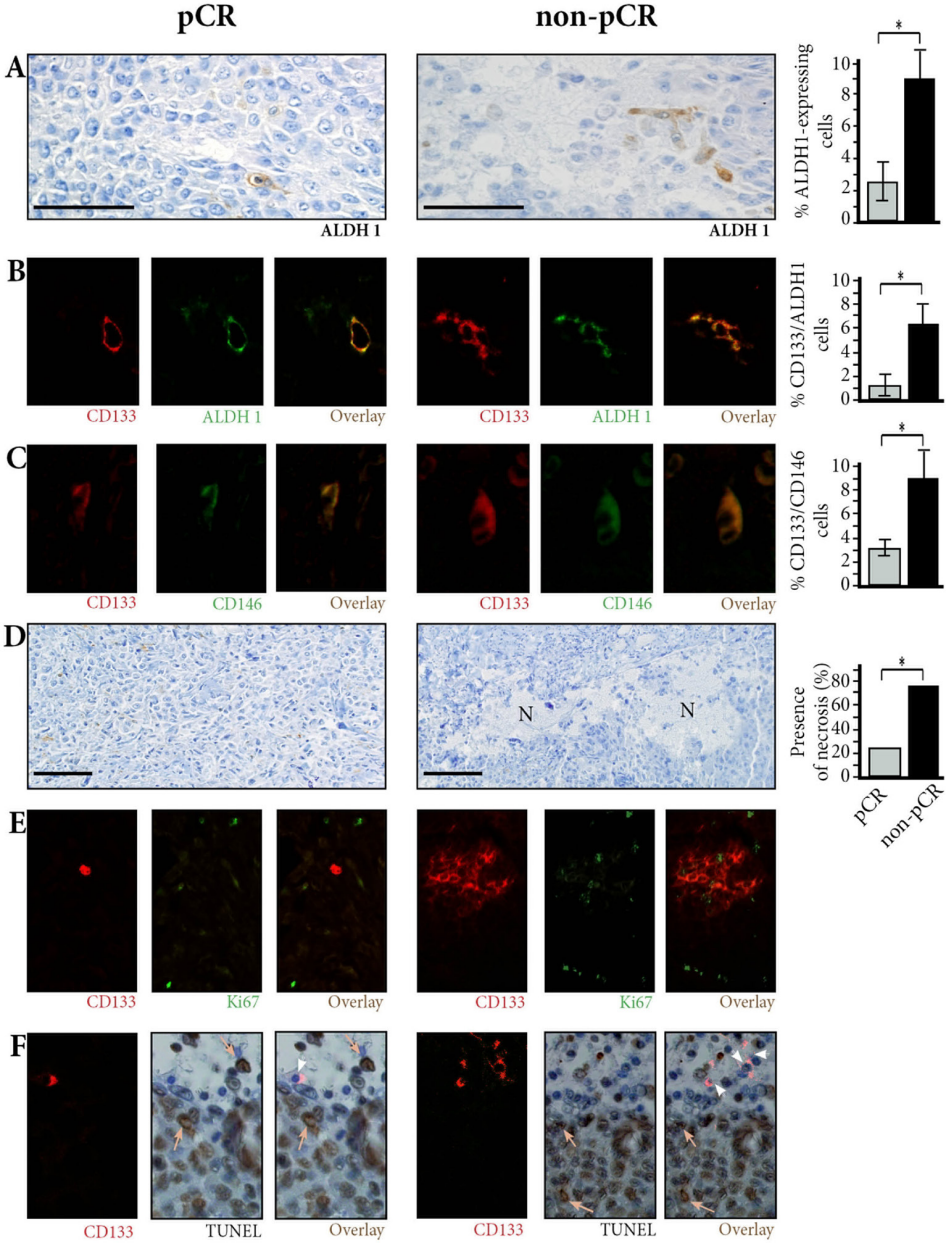
Breast cancer stem-cells in pre-treatment biopsies (**A**) ALDH1-expressing cells are few in pCR patients, more numerous in non-pCR patients. Immunoperoxydase ×400. (**B**) Co-expression of CD133 and ALDH1 markers is found in tumor cells. Double immunofluorescence (IF) ×800. (**C**) Co-expression of CD133 and CD146 markers is found in tumor cells. Double IF ×800. (**D**) Small areas of necrosis (N) are found in non-pCR patients. ×200. (**E**) Ki67-expressing cells do not co-express CD133 except for one cell in the non-pCR patient. Double IF ×400. (**F**) CD133-expressing cells have blue, negative nuclei on TUNEL assay (arrowheads), contrasting with characteristic brown, apoptotic nuclei (arrows). Combined CD133 fluorescence labeling and TUNEL assay. ×400.

Counted on single immunoperoxydase staining (Figure 1A), CD133 expressing cells, ALDH1 expressing cells as well as CD146 expressing cells were significantly more numerous in non-pCR versus pCR patients (10.4% vs. 3.5%, *p <* 0.01; 8.6% vs. 2.6%, *p <* 0.01, and 17.8% vs. 6.3%, *p <* 0.01 respectively).

Since CD133, ALDH1, and CD146 might not precisely identify the same stem-cells, we performed double immunofluorescence stainings and counted the cells co-expressing CD133 and ALDH1 (Figure 1B), and the cells co-expressing CD133 and CD146 (Figure 1C). In the 78 biopsies, the number of CD133/ALDH1 coexpressing cells and the number of CD133/CD146 coexpressing cells were again significantly larger in non-pCR patients than in pCR patients (6.3% ± 1.2 *vs*. 1.2% ± 0.3 for CD133/ALDH1 coexpressing cells, and 9.2% ± 3.1 vs. 3.1% ± 1.1 for CD133/CD146 coexpressing cells) (*p <* 0.01 in both cases).

### Breast cancer stem-cells are in a quiescent autophagic state related to hypoxia in patient tumor samples

As biopsies had been performed at some distance from necrotic areas detectable on ultrasonography, necrosis, when present, was restricted to small areas of mean 0.37 mm<sup>2</sup> (± 0.14 mm<sup>2</sup>) (Figure 1D). Necrosis being an indirect marker of hypoxia [5], we assessed presence or absence of necrosis on the biopsies. Presence of necrosis was significantly associated with non-pCR (*p <* 0.01), and with the number of CD133/ALDH1 or CD133/CD146 co-expressing cells (*p <* 0.01).

We then assessed proliferation and apoptosis in patient tumor samples (Figure 1E and 1F, Supplementary Table 1). When all tumor cells were counted, the Ki67 proliferation rate and apoptotic cell numbers were not significantly different in pCR and non-pCR patients (41% versus 32.3%, *p* = 0.14; and 24% versus 20.5%, *p* = 0.21 respectively). When only CD133 expressing cells were counted in non-pCR patients, proliferation and apoptosis rates were both significantly lower than in all tumor cells (*p <* 0.05).

**Figure 2:**
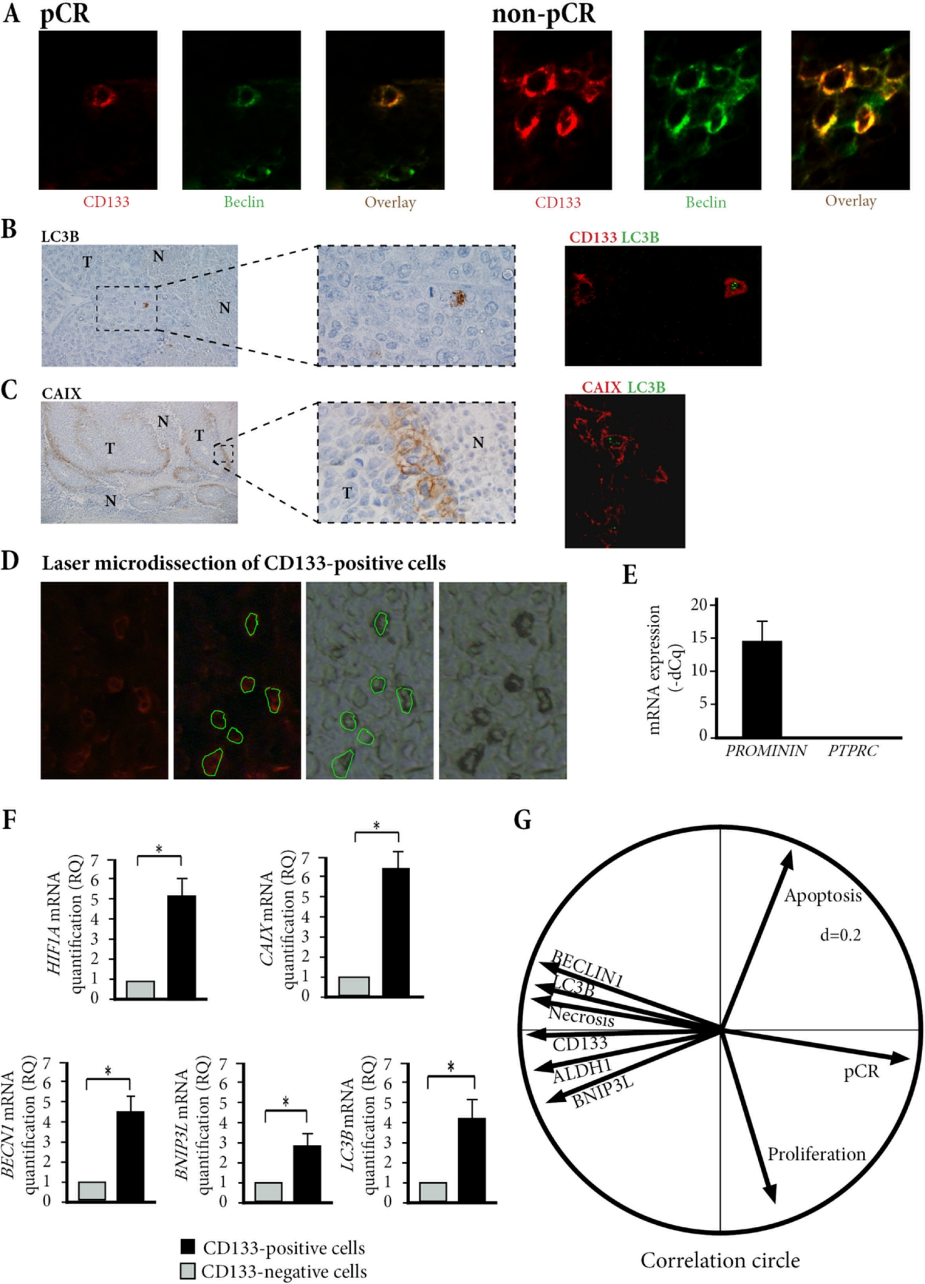
Hypoxia and autophagy markers in breast cancer stem-cells (**A**) Co-expression of CD133 and BECLIN1 in pCR and non-pCR patients. Double IF X800. (**B**) Tumor cells (T) distributed around necrosis (N) express LC3B with typical cytoplasmic punctuated staining, ×1000. Double IF shows that some CD133-positive tumor cells co-express LC3B. ×400. (**C**) Tumor cells (T) distributed around necrosis (N) express CAIX, on their cytoplasmic membrane, ×1000. Double IF shows a co-expression of LC3B and CAIX in some tumor cells. ×400. (**D**) Laser microdissection of CD133-positive cells. (**E**) Laser-microdissected CD133-positive cells express *PROMININ* and not *PTPRC* (CD45). (**F**) mRNA relative quantification of *HIF1A*, *CAIX*, *LC3B*, *BECN1*, and *BNIP3L* show a significantly higher expression level in CD133-positive cells versus CD133-negative cells, in the 35 non-pCR biopsies. *p < 0.05. (**G**) Multivariate analysis of tumor cell characteristics and pCR shows an inverse correlation between pCR and cells bearing markers of stemness or autophagy.

To determine if this quiescent state of breast cancer stem cells was linked to activation of autophagy pathway, we assessed the expression of three autophagy related proteins: BNIP3L, BECLIN1, and LC3B. Particularly, for LC3B marker which enables the detection of autophagic vesicles [20, 21], we observed typical cytoplasmic punctuated staining (Figure 2B), as previously reported [22]. When all tumor cells were counted, numbers of BECLIN1 expressing cells, BNIP3L expressing cells, and LC3B expressing cells were significantly smaller in pCR patients than in non-pCR patients (1.1% *vs*. 5.8%, 8.5% *vs*. 18%, and 3.6% *vs*. 12% respectively, *p <* 0.05).

In CD133-expressing cells, the percentage of cells with autophagic activity was significantly higher than in all tumor cells, of 68%, 79% and 75% for BECLIN1, BNIP3L, and for LC3B respectively (*p <* 0.01) (Figure 2A, 2B and Supplementary Table 1). We checked a possible link between hypoxia and autophagy marker expression in these breast cancer stem-cells. On pre-treatment biopsies, tumor cells expressing carbonic anhydrase IX (CAIX), a hypoxic-related protein [23], were distributed in peri-necrotic areas (Figure 2B). In addition, double immunostaining showed that LC3B-expressing cells co-expressed CAIX (Figure 2C), and were thus preferentially distributed in the peri-necrotic areas. To control these results, we used laser-microdissection of CD133-expressing and CD133-negative tumor cells in pre-treatment biopsies of non-pCR patients (Figure 2D and 2E). We found that CD133-expressing stem-cells had significantly higher mRNA expression levels of *BECN1*, *BNIP3L, MAPLC3B, CAIX* and *HIF1A* than CD133-negative tumor cells (*p* < 0.05) (Figure 2F).

### Multivariate analysis

Cancer stem-cell numbers, presence of necrosis, and numbers of Ki67-, TUNEL-, BECLIN1- BNIP3L-, or LC3B-expressing cells were studied in multivariate analysis for their correlation with pCR. Multivariate forward regression showed that CD133, BECLIN1, BNIP3L and LC3B expression was negatively associated with pCR (*p* < 0.05) and the principal component analysis also assessed this negative correlation (Figure 2G).

Taken together, breast cancer stem-cells in pre-treatment biopsies of TNBC patients with non-pCR after neoadjuvant chemotherapy were neither proliferative nor apoptotic, but in an autophagic state related to hypoxia.

### Patient-derived xenografts are relevant pre-clinical pharmacological models

**Figure 3:**
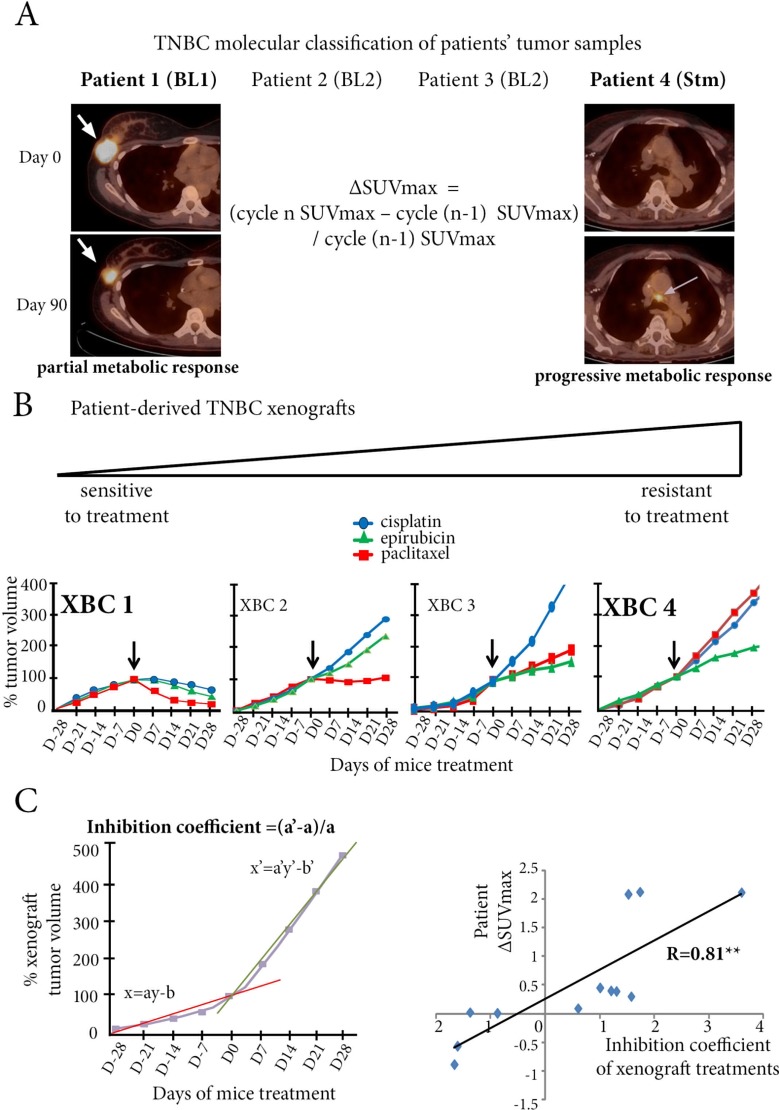
Patient-derived TNBC xenografts are relevant preclinical pharmacological models (**A**) Patients 1, 2, 3 and 4 have breast cancers with basal-like 1 (BL1), basal-like 2 (BL2), BL2, and stem-like (Stm) molecular types respectively. Patient 1 (left panel) has a breast lesion (white arrow) on fused PET/CT with intense fluoro-deoxyglucose uptake at Day 0 and a marked decrease in SUVmax at Day 90 after 2 cycles of epirubicin-cyclophosphamide. In contrast (right panel), a subcarinal hypermetabolic lymph node (grey arrow) appears at Day 90 in Patient 4, after 2 cycles of paclitaxel and bevacizumab. (**B**) Corresponding patient-derived xenografts obtained from tumor samples from these four patients. Treatment with cisplatin, epirubicin, or paclitaxel identifies two opposite models, one chemosensitive (XBC1, derived from Patient 1), the other chemoresistant (XBC4, derived from Patient 4). XBC2 and XBC3 models have an intermediate profile of drug sensitivity. (**C**) In all xenograft models, the coefficient of inhibition for a drug is calculated as (a’-a)/a, a being the slope of the curve before the start of treatment (Day 0), and a’ the slope of the curve between Day 0 and Day 28 of treatment. The right hand panel shows a significant correlation (*R* = 0.81) between ΔSUVmax of a patient chemotherapy regimen with the coefficient of inhibition of the corresponding xenograft with the same chemotherapy. **p < 0.01.

Four patient-derived xenograft murine models (XBC1 to XBC4), previously obtained from samples of four metastatic TNBC, were classified from transcriptomic analyses as basal-like 1 (BL1), basal-like 2 (BL2), and stem-like (Stm) (Supplementary Excel file, Supplementary Table 2, and Figure 3A). The molecular signature of each xenograft model remained unchanged over the 10 successive passages used in this study.

Each xenograft model was tested for three cytotoxic drugs used for patients with metastatic TNBC: epirubicin, paclitaxel, and cisplatin. Of the four models, one, XBC1, was chemosensitive to the three drugs, and one, XBC4 corresponding to the stem-like tumor, was chemoresistant. XBC2 and XBC3 xenograft models had intermediate sensitivity to the three drugs (Figure 3B).

Each individual xenograft was also tested for drugs used for the corresponding patient during her clinical course (Supplementary Table 3). For each chemotherapy tested in mice, an inhibition coefficient was calculated (Figure 3C), using a ratio of the slopes (a and a’) of the straight lines before and after treatment. We found a strong correlation between the ΔSUVmax of a chemotherapy in a patient and the inhibition coefficient in the corresponding xenograft with the same chemotherapy (*R* = 0.81, *p <* 0.01) (Figure 3C).

Overall, our xenograft models from human metastatic TNBC were relevant pre-clinical pharmacological models to study chemoresistance.

### Experimental hypoxia induced autophagy and resistance to drugs in cancer stem-cells derived from the sensitive TNBC xenograft model

Further experiments were performed using XBC1 chemosensitive and XBC4 chemoresistant patient-derived xenograft models.

When we assessed CD133, ALDH1, and CD146 expression in the two models, the numbers of cells expressing CD133 or CD146 were significantly larger in XBC4 than in XBC1. As in patients, CD133-expressing cells did not co-express Ki67 and were negative on TUNEL assay (Supplementary Figure 2).

**Figure 4:**
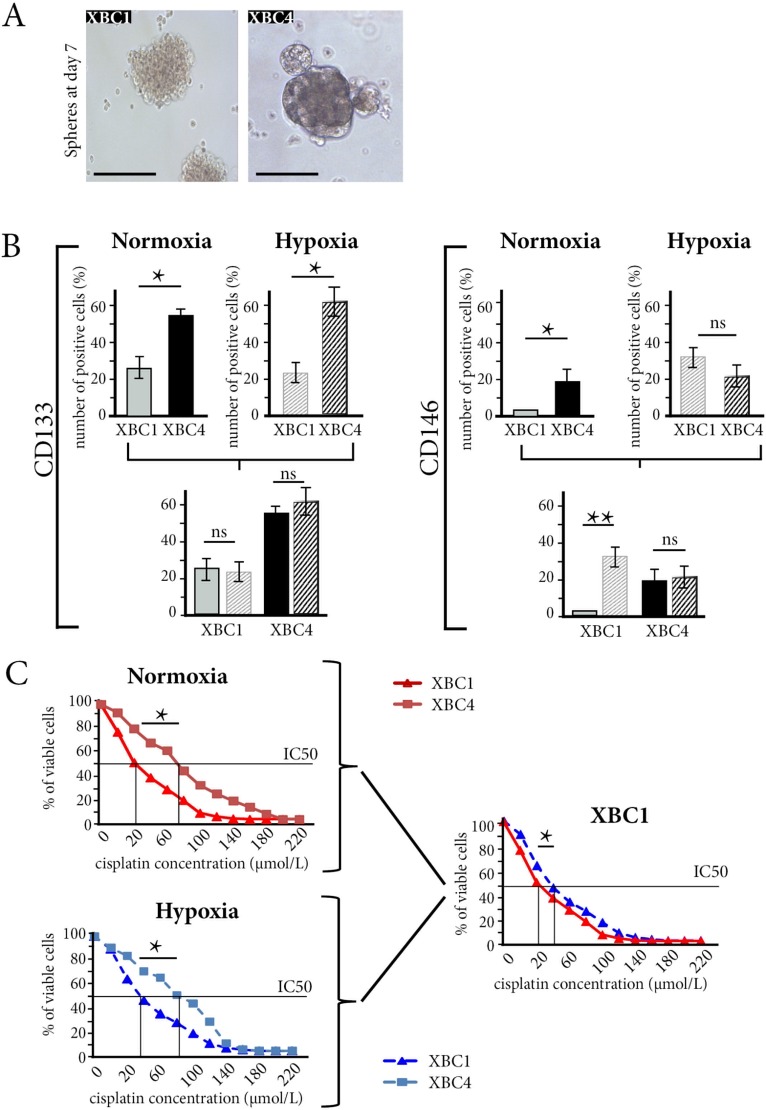
Experimental hypoxia induces autophagy and drug resistance in cancer stem-cells derived from XBC1, the sensitive TNBC xenograft (**A**) Spheres from XBC1 and XBC4 at Day 7 after sorting of CD133-expressing cells from tumors. ×40. (**B**) Under normoxia, the number of CD133- or CD146-expressing cells is larger in XBC4 spheres than in XBC1spheres. Hypoxia increases the number of CD146-expressing cells in XBC1, but not in XBC4. *p < 0.05, **: *p <* 0.01, ns = not significant. (**C**) Under normoxia, XBC1spheres have a higher sensitivity to cisplatin than XBC4 spheres. Hypoxia increases drug resistance of XBC1 spheres. *p < 0.05, IC = inhibitory concentration

We grew spheres larger than 100 μm diameter [24] from CD133-positive cells sorted from XBC1 and XBC4 models (Figure 4A). Under normoxic conditions, CD133-expressing cells and CD146-expressing cells were more numerous in XBC4 spheres than in XBC1 spheres (*p <* 0.05) (Figure 4B).

When XBC1 spheres were cultured under hypoxic conditions, the number CD146-expressing cells increased from 3 to 31% (*p <* 0.01), a high percentage comparable to the 20% of CD146-expressing cells in XBC4 spheres (Figure 4B).

For autophagy markers, in normoxia, mRNA *BECN1* and *BNIP3L* levels were overexpressed in XBC4 spheres compared to XBC1 spheres (*p <* 0.05). Experimental hypoxia increased *BECN1* and *BNIP3L* mRNA levels in both XBC1 and XBC4 spheres (*p <* 0.05) (Supplementary Figure 3).

Under normoxic conditions, resistance to cisplatin, epirubicin and paclitaxel was higher for XBC4 spheres than for XBC1 spheres. Experimental hypoxia increased resistance to the three drugs in XBC1 spheres, but not in XBC4 spheres (Supplementary Table 4, Figure 4C for cisplatin).

### *In vitro* autophagy inhibition reversed chemoresistance in cancer stem-cells derived from the resistant TNBC xenograft model

**Figure 5:**
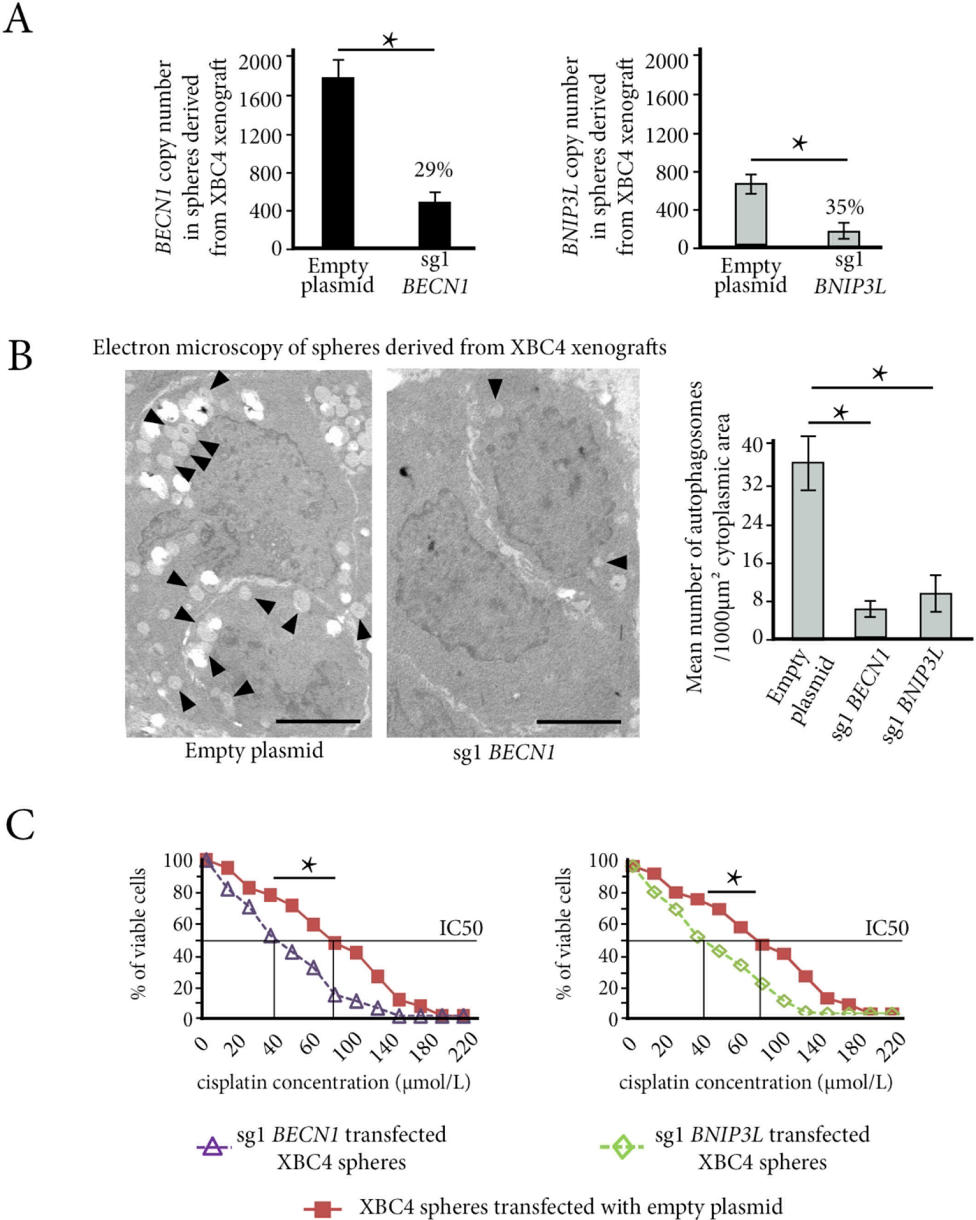
*In vitro* autophagy inhibition reverses chemoresistance in cancer stem-cells derived from XBC4, the resistant TNBC xenograft model (**A**) Invalidation of *BECN1* or *BNIP3L* genes with CRISPR-Cas9 technology in XBC4 spheres. Bar graphs show a decrease of 71% in *BECN1* copy number in sg1*BECN1* transfected cells (left panel), and a decrease of 65% in *BNIP3L* copy number in sg1*BNIP3L* cells (right panel). (**B**) The mean number of autophagosomes per 1000 μm<sup>2</sup> of cytoplasmic area on electron microscopy is lower in XBC4 spheres transfected with sg1*BECN1* or with sg1*BNIP3L* than in XBC4 spheres transfected with empty plasmids. (**C**) Under normoxia, spheres from XBC4 transfected with sg1*BECN1* or with sg1*BNIP3L* have a higher sensitivity to cisplatin than spheres transfected with empty plasmids. *p < 0.05.

To understand the role of autophagy in chemoresistance of breast cancer stem cells, we invalidated *BECN1* or *BNIP3L* expression in XBC4 spheres. To knock out these two genes, we used the innovative CRISPR-CAS9 technology, particularly suited to spheres with brief viability (Supplementary Figure 4). 48 h after transfection and antibiotic selection, we obtained significant decreases in mRNA copy numbers: 71% (from 1798 to 525, *p <* 0.05) for *BECN1* and 65% (from 683 to 239, *p <* 0.05) for *BNIP3L* (Figure 5A).

Electron microscopic study focused on cytoplasmic areas of tumor cells (see Methods and Supplementary Figure 5). It showed a striking difference between tumor cells transfected with empty plasmid, which had large numbers of cytoplasmic autophagosomes, and sg1*BECN1* or sg1*BNIP3L* transfected tumor cells (Figure 5B).

Counts performed on cytoplasmic areas of 25 cells in each experimental condition showed that the mean number of autophagosomes per 1000 μm<sup>2</sup> of cytoplasmic area was 35.8 in cells transfected with empty plasmid *versus* 6.5 and 9.8 in sg1*BECN1* and sg1*BNIP3L* transfected cells respectively (*p <* 0.05 in both cases).

Resistance to cisplatin, epirubicin and paclitaxel significantly decreased in XBC4 spheres transfected either with sg1*BECN1* or sg1*BNIP3L* when compared to XBC4 spheres transfected with empty plasmid (illustrated for cisplatin in Figure 5C, Supplementary Table 5).

### *In vivo* autophagy inhibition reversed chemoresistance in resistant TNBC xenograft model

**Figure 6:**
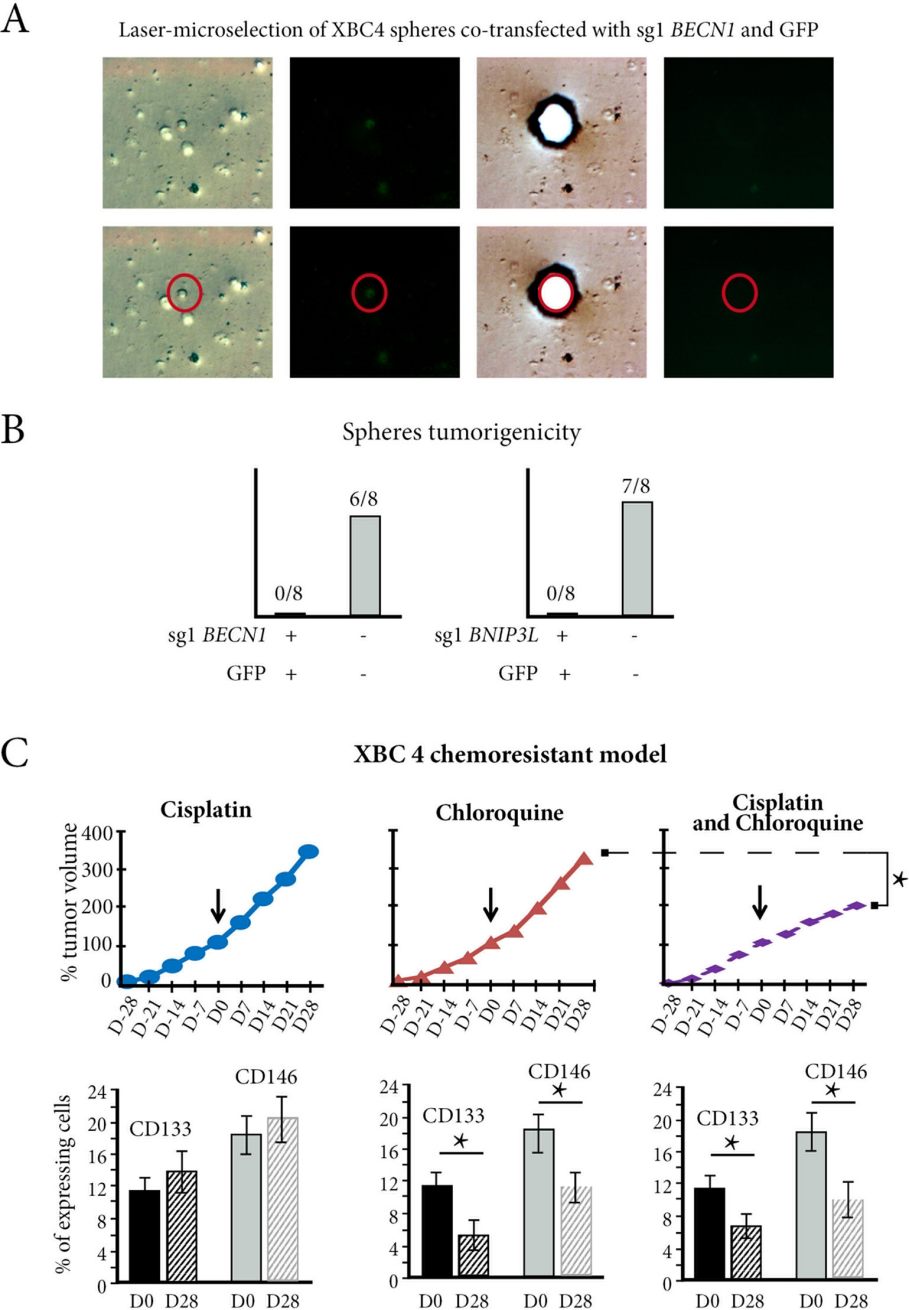
*In vivo* autophagy inhibition reverses chemoresistance in resistant TNBC xenograft (**A**) Cells from XBC4 spheres co-transfected with sg*BECN1* and GFP have green fluorescence, enabling a single cell laser-microdissection (red cycle). (**B**) GFP positive-cells (GFP+) co-transfected with sg*BECN1* or with sg1*BNIP3L* are not tumorigenic, while GFP-negative cells are. (**C**) Treatment of the XBC4 chemoresistant model with chloroquine does not inhibit tumor growth at Day 28, but the numbers of CD133 or CD146-expressing cells decrease. Chloroquine added to cisplatin inhibits tumor growth when compared to chloroquine alone. It also induces a decrease in CD133 or CD146-expressing cell numbers when Day28 is compared to Day 0. *p < 0.05.

We assessed the tumorigenicity of XBC4 spheres co-transfected with GFP and sg1*BECN1* or sg1*BNIP3L*. Using laser micro-dissection in liquid medium, GFP-positive cells were selected 48 h after transfection and antibiotic selection (Figure 6A). We checked that these cells were viable and that they also had a more than 65% decrease in *BECN1* or *BNIP3L* copy numbers, but when 200 cells were injected sub-cutaneously in 8 mice, there was no engraftment (Figure 6B). Conversely, when we selected GFP-negative cells, they had no significant decrease in *BECN1* or *BNIP3L* copy numbers, and 200 cells were tumorigenic in 6/8 and 7/8 grafted mice respectively (Figure 6B).

When we used an autophagy inhibitor, chloroquine [25], to treat the three cisplatin-resistant xenografts, XBC2, XBC3 and XBC4 (Figure 6C and Supplementary Figure 6), there was no change in tumor growth at Day 28, but the numbers of CD133 or CD146-expressing tumor cells significantly decreased (*p <* 0.05) (illustrated for XBC4 in Figure 6C).

When chloroquine was added to cisplatin, there was significant tumor growth inhibition compared to treatment with chloroquine alone in the three cisplatin-resistant xenografts: the percentage of tumor volume at Day28 was 267% *versus* 365% for chloroquine alone (*p <* 0.01) for XBC2; 208% *versus* 432% for chloroquine alone (*p <* 0.01) for XBC3; 202% *versus* 345% for chloroquine alone (*p <* 0.01) for XBC4 (Supplementary Figure 6).

The combination of cisplatin and chloroquine in the three cisplatin-resistant xenografts also induced a significant decrease in the numbers of CD133 or CD146-expressing cells when Day28 was compared to Day 0 (*p <* 0.01) (illustrated for XBC4 in Figure 6C).

We thus demonstrated that the molecular or chemical inhibition of the autophagic pathway was able to reverse chemoresistance in triple negative breast carcinoma.

## DISCUSSION

In this study, we demonstrated the role of hypoxia and autophagy in the resistance to chemotherapy of cancer stem-cells in TNBC.

In pre-treatment biopsies of 78 patients with TNBC, we first showed that chemoresistance was associated with large numbers of cancer stem-cells. These cancer stem-cells were hypoxic, and this was not due to tissue processing since all pre-treatment biopsies were performed under ultrasonography to avoid large necrotic areas. Biopsies were calibrated and processed identically, enabling us to analyze comparable areas of tumor tissue. [26] After chemotherapy, pathological response was assessed on breast surgery pieces. The pCR rate we found was 25.6%, close to the 27.9% pCR rate reported in 663 TNBCs treated with neoadjuvant chemotherapy. [10]

For *in situ* characterization of breast cancer stem cells, we did not use the combination of markers CD44^+^CD24^−/low^, [18, 27] since this combination of positive and negative markers, currently used in cytometry, is more difficult to translate into *in situ* tissue analyses and cell counts. [28] CD133, ALDH1 and CD146 are three positive markers used for studying triple negative breast cancer stem-cells. [29–32] In addition, CD133 and ALDH1 cancer stem cells are preferentially distributed in hypoxic areas [5, 33] while CD44^+^CD24^−/low^ breast cancer stem cells are mainly found on the tumor invasive edge. [28] In the pre-treatment biopsies studied here, whether counted on single staining or on combined double staining, breast cancer stem-cell numbers were significantly larger in non-pCR patients than in pCR patients. This could result from changes in proliferation and/or apoptosis rates. But the Ki67 proliferation rate was significantly lower for stem-cells than for tumor cells as a whole, a result coherent with *in vitro* studies showing that ALDH1-positive breast cancer stem-cells are not in a proliferative state. [34] In the same pre-treatment biopsies, the apoptosis rate was also significantly lower for stem-cells than for tumor cells as a whole. This fact, not hitherto reported, could contribute to the maintenance of a large number of cancer stem-cells in non-pCR patients.

The role of autophagy in the quiescent state of cancer stem-cells [2] and in the maintenance of breast cancer stem-cells [18, 19, 27, 35] has recently been demonstrated. When we looked for autophagy markers in our pre-treatment biopsies of non-pCR TNBCs, we found that CD133-expressing cancer stem cells co-expressed BECLIN1, LC3B, and BNIP3L. When we used another independent method based on laser-microdissection of CD133-expressing cells combined with quantitative PCR, we also found an expression of the three autophagy markers in cancer stem-cells. A multivariate analysis based on cell counts found an inverse correlation between the number of cells expressing markers of stemness or autophagy and pCR, a bio-clinical correlation hitherto not demonstrated.

On the basis of our experience with patient-derived renal cancer xenograft for the study of cancer stem-cells and resistance to drugs, [5] we implemented patient-derived xenografts of TNBC. Patient-derived xenografts are more relevant pharmacological tools than xenografts from human cancer cell lines, which have poor reliability in predicting response to chemotherapy in patients. [36–38] Using four patient-derived xenograft models with genomic stability over passages, we found that three of them had resistance to cisplatin. Two of the four models had opposed sensitivity to three drugs used to treat women with metastatic TNBC. These two models reproduced the clinical response to drugs, with a large number of quiescent cancer stem-cells in the chemoresistant model. We focused our pre-clinical studies on breast cancer stem-cells derived from these two opposite models to study the mechanisms of drug resistance in TNBC stem-cells.

We first demonstrated that hypoxia increased drug resistance of autophagic TNBC stem-cells. Using spheres derived from the chemosensitive model, we showed that experimental hypoxia increased the number of autophagic cancer stem-cells, a result in accordance with *in vitro* studies using human cancer cell lines. [19, 39, 40]

In the same chemosensitive model, experimental hypoxia also increased resistance to cisplatin, epirubicin, and paclitaxel. So far, a link between hypoxia and drug resistance of cancer stem-cells has only been demonstrated for anti-angiogenic drugs. Sunitinib generates tumor hypoxia and increases the numbers of cancer stem-cells in xenografts obtained from human TNBC cell lines. [33] In human renal carcinomas, we demonstrated that sunitinib was able to generate resistance to its own therapeutic effect *via* induced hypoxia in cancer stem cells. [5] Here, on TNBC cancer stem-cells, we studied three cytotoxic drugs with main effects not on angiogenic vessels but on tumor cells. Cisplatin forms platinum adducts with DNA, which impedes DNA replication and transcription, thus leading to apoptotic cell death. [41] Paclitaxel is a taxoid that binds to tubulin polymers and inhibits microtubule depolymerisation, thus disrupting tumor cell microtubule dynamics and leading to apoptotic cell death. [42] Epirubicin is an anthracycline that inhibits the activity of topoisomerase II, thus impairing tumor cell DNA replication. However, a recent study demonstrated that paclitaxel is able to induce the HIF pathway in TNBC cell lines. [43] Thus, although it does not target angiogenic vessels, paclitaxel is able to induce hypoxia in tumoral tissue. The originality of our results is that they link hypoxia, autophagy and drug resistance in TNBC stem-cells. We used experimental hypoxia on TNBC stem-cells to reproduce the results found in biopsies of TNBC patients resistant to chemotherapy who had a large number of stem-cells expressing autophagic markers around necrotic areas.

This link between hypoxia, autophagy and drug resistance in TNBC stem-cells raises a new issue for the translational relevance for women with metastatic TNBC. Is it possible to modulate autophagy in breast cancer stem-cells to reverse resistance to chemotherapy? In recent years, autophagy has been proposed as a potential target for cancer therapy. [25] There are pre-clinical [44, 45] and clinical [46, 47] data on the potential benefit of autophagy inhibition as an anticancer therapy. Particularly, in a pediatric brain tumor with *BRAF* mutation, hydroxycholoroquine was able to reverse acquired resistance to BRAF inhibitor. [48] In our study, we demonstrated both *in vitro* and *in vivo* that autophagy inhibition was able to reverse resistance to drugs. *In vitro*, using spheres derived from the chemoresistant patient-derived xenograft model, the molecular invalidation of autophagy markers enabled us to reverse chemoresistance to cisplatin, epirubicin and paclitaxel. *In vivo*, we used chloroquine, a chemical inhibitor of autophagy, and showed that chloroquine alone induced a decrease in numbers of cancer stem cells, but with limited cytotoxic effect and thus no change in tumor growth inhibition, two effects previously described in *in vitro* models [49, 50]. However chloroquine added to cisplatin was able to reverse resistance to cisplatin in the three cisplatin-resistant patient-derived xenograft TNBC models, probably as a result of autophagy inhibition in the breast cancer stem cells but also in the whole tumor.

This study also affects existing knowledge in the field of response to therapy in TNBC, since it identifies a marker predictive of response to chemotherapy in biopsies performed before any treatment, thus at the optimal moment to design a therapeutic strategy.

The clinical relevance of our results is a major strength of this work, and this has been obtained through rigorous clinical, radiological and pathological methodologies. However, the generalisation of the conclusions requires a validation cohort.

## MATERIALS AND METHODS

### Patients and tumor samples

In the Centre-Maladies-du-Sein, Hôpital-St-Louis, Paris, a registry of patients treated with neoadjuvant chemotherapy for localized breast carcinoma prospectively enrolled 361 patients between 2005 and 2011. For diagnostic purposes, all patients underwent core needle biopsies before chemotherapy; 107 had ductal triple negative adenocarcinoma (TNBC).

Ultrasonography-guided pre-treatment biopsies were similarly performed, using a 16-gauge needle, at least 1 cm from necrotic areas detected on ultrasonography. Tissue samples were similarly processed, one formaldehyde-fixed and paraffin-embedded, and one snap-frozen.

In compliance with French Bioethics law (2004–800, 06/08/2004), all patients had been informed of the research use of the part of their samples remaining after diagnosis had been established, and did not oppose it. Informed consent was obtained for each patient. Tumor tissue was available for this study for 78 of the 107 TNBC patients.

Surgical pieces obtained after neoadjuvant chemotherapy were independently analyzed by two pathologists (AR, PB) unaware of response to treatment. pCR was defined, according to [10], by absence of residual tumor at time of surgery both in breast and axillary lymph node samples.

Radiotherapy was delivered after surgery to patients who had breast-conserving surgery or mastectomy for initial advanced stages. The patients’ follow-up was clinical, biological and radiological according to institutional guidelines, and the relapse rate was calculated considering local and metastatic relapse, or cancer-related death.

### Necrosis assessment in human tumor samples

The 78 pre-treatment biopsies were reviewed by two pathologists unaware of response to treatment (AJ, CL) for the presence or absence of necrotic areas. When present in any field of the two different sections analyzed for each biopsy, necrosis was delineated on virtual slides created on a Nanozoomer2.0H scanner (Hamamatsu/Japan), and quantified using DotSlide2 software. Results were expressed as the sum of necrotic areas for each biopsy, and the mean ± SEM.

### Cancer stem-cell characterization and counts in human tumor samples

CD133, ALDH1 and CD146 expression was assessed and cells counted on TNBC pre-treatment biopsies; we also counted ALDH1/CD133 and CD146/CD133-coexpressing cells (Supplementary Methods).

### Proliferation, apoptosis, autophagy and cancer stem-cells in human tumor samples

On pre-treatment biopsies, i) tumor cell and cancer stem-cell proliferation rates were assessed; ii) *in situ* apoptosis was assessed using TUNEL assay, and CD133-expressing/TUNEL-expressing cells were counted; iii) the expression of three cellular markers of autophagy, BECLIN1, BNIP3L, and LC3B was assessed (Supplementary Methods).

### Autophagy and hypoxia in cancer stem-cells of human tumor samples

To characterize hypoxic areas on non-pCR pre-treatment biopsies, we assessed the *in situ* expression of a hypoxic-related protein, CAIX.

To check any possible co-expression of hypoxic and autophagy markers in cancer cells, we performed double immunofluorescent stainings using anti-human LC3B and CAIX (NB100-417, Novus Biologicals, France) antibodies, labelled with Apex-alexa-Fluor 488 nm and 647 nm (A10475, Life-Technology) respectively.

To control these immunostaining results, we used a completely different method combining morphological analyses and molecular markers. On frozen pre-treatment biopsies, CD133-expressing cells were laser-microdissected and their *BNIP3L, BECN1, MAP1LC3B, CAIX* and *HIF1A*-expression levels assessed using quantitative PCR (Supplementary Methods).

### Patients with metastatic TNBC and gene expression profiling

Four women with metastatic TNBC, different from the 78 other patients, participated in this study.

For each patient, five samples of a metastasis were obtained during the procedure of imaging-guided biopsy at the time of relapse, before any medical treatment. Informed written consent was obtained from the patients. The Clinical Research Board Ethics Committee approved this study (CPP Ile-de-France N°13218).

Among the five tumor samples, i) two were formaldehyde-fixed and paraffin-embedded for histological analyses, ii) two were immediately snap-frozen in liquid nitrogen and stored in Hôpital-St-Louis Tumorbank for molecular analysis, iii) and one was set aside in culture medium for xenografting.

Total RNA was extracted from the frozen tumor sample as above, and transcriptomic analyses were performed using MiltenyiBiotec Microarray (Supplementary Methods). Classification was provided by correlating gene expression profiles with the centroids for each of the 6 TNBC subtypes described by Lehmann et al [51], and with Parker centroids for PAM50 classification ([52], https://genome.unc.edu/pubsup/breastGEO/pam50_centroids.txt).

### Patient-derived breast cancer xenografts and treatments

Four patient-derived xenografts of human TNBC were studied (XBC1 to XBC4). They had been established for a pilot study on personalized treatment for women with metastatic triple negative breast carcinoma [53], before any medical treatment of the metastatic disease (Supplementary Methods).

The University Institute Board Ethics Committee for experimental animal studies approved this study (N°2012-15/728-0115).

For each xenograft model, the day when tumors reached a volume of 300mm3 – i.e. 100% tumor volume – was considered as Day0. Mice (*n* = 5 mice per treatment group) were then treated over 28 days with three types of chemotherapy (Supplementary Table 6 for drugs tested in TNBC xenografted mice). A daily clinical score was recorded and tumor growth was measured weekly until tumor weight reached the ethically recommended limit of less than 10% of mouse weight (Directive 2010/63/EU of European Parliament and Council of 22 September 2010 on the protection of animals used for scientific purposes; Official Journal of European Union L 276/33).

### Assessment of tumor response in patients

For each line of chemotherapy, the patient response under treatment was characterized (Supplementary Methods).

### Cancer stem-cells from xenografted human TNBC: spheres and cytotoxicity

We studied spheres obtained from untreated tumor samples of XBC1 and XBC4 xenograft models.

Cells expressing CD133 were obtained from dissociated sections using magnetic sorting, with anti-CD133 micro-bead antibody (Miltenyi-Biotech, Germany), using the manufacturer's procedures. We controlled CD133 cell purity by flow cytometry. CD133 positive cells were placed in a low-attachment six-well plate at a density of 2000 to 5000 cells/well, and cultured in a serum-free, high-glucose medium (DMEM-F12, Gibco, France) supplemented with 2% B27-NeuroMix and 0.4% fetal-bovine-serum (PAA, France), 5 mg/mL insulin (Sigma, France), 20 ng/mL epidermal-growth-factor (R&D systems, France), and 0.5 ng/mL hydrocortisone (Sigma, France). Spheres were obtained after 48 h of culture in a humidified chamber (37°C, 5% CO_2_) under normoxia (20% O_2_) for further *in vitro* functional experiments and *in vivo* tumorigenicity experiments (Supplementary Figure 4). For cytotoxicity tests, see Supplementary Methods.

### Experimental hypoxia, assessment of stem-cell markers and of mammosphere sensitivity to drugs

For the two xenograft models, XBC1 or XBC4, spheres were separated into two groups maintained in a humidified chamber for 48 h, one under experimental hypoxia (1% O2) and the other under normoxia (20% O2). For experimental hypoxia, we chose a pO2 level of 1% as it is now accepted that stem-cell niches are hypoxic with oxygen tension as low as 1% [54].

The relative number of CD133 and CD146 expressing cells in hypoxic and normoxic spheres was assessed by flow cytometry, and for cytotoxicity, MTT test was performed (Supplementary Methods).

### Experimental hypoxia and autophagy in spheres

Total RNA was extracted from normoxic and hypoxic spheres. TaqMan RT-qPCR was performed to determine *BECN1* and *BNIP3L* gene expression levels.

### Knock-out of autophagy gene expression in spheres

Because of a 16-day lifetime of spheres derived from patient-derived xenografts (XBC1 and XBC4 models), CRISPR-CAS9 technology was chosen to invalidate *BECN1* and *BNIP3L* gene expression. A dedicated algorithm (seehttp://crispr.mit.edu website) enabled us to choose the target sequence of 20 bases around the active sites of phosphorylation for each of the two genes. Oligos designed to build the vectors containing the sgRNA1 targeting *BECN1* or *BNIP3L* genes are detailed in Supplementary Table 7, and the method in Supplementary Methods.

### Droplet digital PCR

Droplet digital PCR was performed to assess the copy number of *BECN1* or *BNIP3L* genes in spheres transfected with empty plasmid and spheres transfected with sg1*BECN1* or sg1*BNIP3L* (Supplementary Methods).

### Electron microscopy of spheres KO for BECN1 or BNIP3L

Spheres transfected with empty plasmid and spheres transfected with sg1*BECN1* or sg1*BNIP3L* were dissociated and cells in suspension were counted. After centrifugation, a pellet of 10^6^ cells was further processed for electron microscopy (Supplementary Methods). Ultrastructural analysis focused on the cytoplasms of tumor cells to detect characteristic autophagosomes according to the “Guidelines for the use and interpretation of assays for monitoring autophagy” [11].

A quantitative study was performed comparing the numbers of autophagosomes per cytoplasmic area in cells transfected with empty plasmid versus sg1*BECN1* or sg1*BNIP3L* transfected cells (Supplementary Figure 5).

### Tumorigenicity of spheres KO for BECN1 or BNIP3L

48 h after co-transfection with GFP and after antibiotic selection, a volume of 10μL of culture medium containing 2 × 10^5^ cells dissociated from spheres was transferred and spread on a specific plate dedicated to laser-microdissection of living cells. Laser-microdissection was performed using a PALM-Microbeam/Zeiss-system equipped with a 480 nm wavelength filter. GFP-positive fluorescent cells were catapulted one by one into a cap with culture medium. GFP-negative cells were also selected using the same process. For a given set of cells, the procedure did not exceed 30 minutes. Selected cells were rinsed, and a minimum of 200 GFP-expressing cells were grafted subcutaneously in nude mice to assess the tumorigenicity of cells transfected with sg1BECN1 or sg1BNIP3L. Cells transfected with empty plasmid served as the control. Mice were followed up over a period of 6 months in dedicated animal housing.

### Chemical inhibition of autophagy in XBC4 xenograft

When tumors reached a volume of 300mm^3^, the mice were treated for 28 days with cisplatin alone, chloroquine alone (50 mg/kg/day intra-peritoneally, Sigma-Aldrich, France), or a combination of cisplatin and chloroquine (*n* = 5 mice per treatment group) (Supplementary Table 8 for drugs tested in TNBC xenografted mice). A daily clinical score was recorded and tumor growth was measured weekly.

### Statistics

The statistical analyses were performed using R 2.15.2 statistical software (R-Foundation for Statistical Computing, Vienna/Austria) (Supplementary methods).

## CONCLUSIONS

Our results support breast cancer stem-cell evaluation in pre-treatment biopsies of TNBC patients, and further research on autophagy inhibition to reverse resistance to chemotherapy.

## SUPPLEMENTARY MATERIALS FIGURES AND TABLES


