# Supplementary material for: Targeting autophagic cancer stem-cells to reverse chemoresistance in human triple negative breast cancer
Source: Oncotarget. 2017 Apr 7;8(21):35205–21. doi: 10.18632/oncotarget.16925 (PMC5471047; doi:10.18632/oncotarget.16925)
Supplement: Supplementary file 1 [file oncotarget-08-35205-s001.pdf]

Targeting autophagic cancer stem-cells to reverse chemoresistance in human triple negative breast cancer

Supplementary Materials

Cancer stem-cell characterization and counts in human tumor samples

CD133, ALDH1 and CD146 expression was assessed on TNBC pre-treatment biopsies using an indirect immunoperoxydase method on 5µm-thick tissue sections, using anti-CD133 (clone AC133/1, MiltenyiBiotec/Germany), anti-ALDH1 (clone44, Becton-Dickinson/France), or anti-CD146 (AB75769, Abcam, UK) as primary antibodies. Systematic controls were absence of primary antibody and use of an irrelevant primary antibody of the same isotype. For each patient, the number of CD133- or ALDH1- or CD146-positive cells was determined on five different fields on the same biopsy. A ProvisAX70 microscope (Olympus/Tokyo) with wide-field eyepiece number 26.5 was used, providing a field size of 0.344mm^2 at x400 magnification. For each field, 100 cancer cells were analyzed by two pathologists (AJ, CL). The percentage of cancer stem-cells was the number of positive cells in these 100 cancer cells. Results were expressed as mean ± SEM.

We also estimated the number of ALDH1/CD133-coexpressing cells, and the number of CD146/CD133-coexpressing cells. Double immunofluorescent staining was performed using anti-human CD133 antibody labelled with Apex-Alexa-Fluor488 Antibody-Labelling-Kit (NY14072,Life-technologies/USA), and anti-human ALDH1 or anti-CD146 labelled with Apex-Alexa-Fluor555 Antibody-Labelling-Kit (Life-technologies). ALDH1/CD133-coexpressing cells and CD146/CD133-coexpressing cells were counted using a double-band epifluorescence filter (U-F51006, Olympus/Tokyo).

Proliferation and cancer stem-cells in human tumor samples

Tumor cell proliferation rate was assessed on pre-treatment biopsies performed before treatment using monoclonal mouse anti-human Ki67 antibody (cloneMIB-1, DakoCytomation/France) as primary antibody. For each biopsy, five different fields at ×400 magnification were analyzed. The proliferation rate was the percentage of positive nuclei in 100 cancer cells analyzed.

To assess cancer stem-cell proliferation, we performed double immunofluorescent staining using the same anti-human Ki67 antibody labelled with Apex-Alexa-Fluor488, and anti-human CD133 antibody labelled with Apex-Alexa-Fluor555. Since CD133-expressing cells were few, the proliferation rate of cancer stem-cells was determined by the ratio of CD133/Ki67-coexpressing cell numbers to CD133-expressing cell numbers. Results were expressed as mean ± SEM.

Apoptosis and cancer stem-cells in human tumor samples

In situ apoptosis was assessed on pre-treatment biopsies using TUNEL assay [1].

Apoptotic cell counts were performed as above for Ki67-expressing cells. The percentage of apoptotic cells in 100 tumor cells was determined, and results expressed as mean ± SEM.

To assess cancer stem-cell apoptosis, TUNEL assay was combined with anti-human CD133 immunostaining on the same tissue section. After TUNEL assay, tissue sections were incubated with mouse monoclonal anti-human CD133 antibody labelled with Apex-Alexa-Fluor555. For each biopsy, five different fields were analyzed. Since CD133-expressing cells were few, the percentage of apoptotic cancer stem-cells was determined by the ratio of CD133-expressing/TUNEL-positive cell numbers to CD133-expressing cell numbers. Results were expressed as mean ± SEM.

Autophagy and cancer stem-cells in human tumor samples

The expression of three cellular markers of autophagy, BECLIN1, BNIP3L, and LC3B was assessed on pre-treatment biopsies. An indirect immunoperoxydase method was performed using rabbit polyclonal anti-BECLIN1 antibody (clone ab16998,Abcam/Cambridge/USA, dilution1:100), or anti BNIP3L antibody (clone ab8399,Abcam, dilution1:50), or rabbit monoclonal anti-LC3B antibody (clone D11, Cell Signaling/France, dilution: 1:3200), [2], as primary antibodies. For each biopsy and each marker, five different fields were analyzed, the percentage of positive cells in 100 cancer cells was determined, and results were expressed as mean ± SEM.

To assess the number of autophagic cancer stem-cells, we performed double immunofluorescent staining using anti-BECLIN1, or anti-BNIP3L, or anti-LC3B

antibody labelled with Apex-Alexa-Fluor488, and anti-human CD133-antibody labelled with Apex-Alexa-Fluor-555. For each biopsy, on five different fields, the autophagic stem-cell percentage was determined by the ratio of the numbers of CD133/BECLIN1, or CD133/BNIP3L, or CD133/LC3B coexpressing cells to the numbers of CD133-expressing cells in each field. Results were expressed as mean ± SEM.

Autophagy and hypoxia in cancer stem-cells of human tumor samples

To characterize hypoxic areas on non-pCR pre-treatment biopsies, we assessed the *in situ* expression of a hypoxic-related protein, CAIX [3, 4].

To check any possible co-expression of hypoxic and autophagy markers in cancer cells, we performed double immunofluorescent stainings using anti-human LC3B, and CAIX (NB100-417, Novus Biologicals, France) antibodies, labelled with Apex-alexa-Fluor 488nm and 647nm (A10475, Life-Technology) respectively.

To control these immunostaining results, we used a completely different method combining morphological analyses and molecular markers. On frozen pre-treatment biopsies, CD133-expressing cells were laser-microdissected and their *BNIP3L, BECN1, LC3B, CAIX* and *HIF1A*-expression levels assessed using quantitative PCR. For each biopsy, a minimum number of 100 CD133-expressing cells was required for molecular analyses, therefore biopsies of only 35 non-pCR patients could be studied. Laser-microdissection was performed using a PALM-Microbeam/Zeiss-system, on 7 µm-thick frozen sections after immunofluorescent staining with anti-CD45 (LCA, Dako/France) antibody. Laser-microdissection was only performed on CD45-negative cells. Using PALM-Robot software for quantification on the 35 biopsies, a mean number of 2000 CD45-negative cells (mean surface 4.10^5 μm^2) were microdissected for each case and catapulted into tubes for RNA extraction.

On following frozen sections, we then performed double immunofluorescent staining with anti-CD133 antibody labelled with Apex-Alexa-Fluor555, and with anti-CD45 (LCA, Dako/France) labelled with Apex-Alexa-Fluor488. Laser-microdissection was only performed on CD133-positive/CD45-negative cells. For each case, a mean number of 200 CD133-positive/CD45-negative cells (mean surface $4.10^4 \mu m^2$) were laser-microdissected.

Total RNA was extracted from microdissected cells using RNeasy-Mini-Kit (Qiagen/Les-Ulis/France), quantified on NanoDrop and qualified on Bio-Rad Experion™ Automated-Electrophoresis-Station (BioRad/Marnes-la-Coquette/France). For RT-qPCR, total RNA was reverse-transcribed using random primers with SuperScript™ II-Reverse-Transcriptase (Invitrogen/Saint-Aubin/France). The qPCR reactions were performed using fluorescent probes on a CFX96 Real Time System (Bio-Rad) to determine *PROMININ1, KRT7, PTPRC, BECN1, BNIP3L*, *MAP1LC3B*, *CA9* and *HIF1A* and gene expression levels, using Hs01009250_m1(*PROMININ1*), Hs00559841_m1(*KRT7*), Hs04189704_m1(*PTPRC*), Hs00186838_m1(*BECN1*), Hs00188948_m1(*BNIP3L*), Hs00797944_s1 (*MAPLC3B*), Hs00154208_m1(*CA9*), Hs00153153_m1(*HIF1A*) as primers (Applied-Biosystem/France). The reference gene was human *TBP* with the primer Hs99999910_m1, a blank sample (no cDNA) was included, and experiments were performed in triplicate, with each sample in duplicate on the PCR plate. The results were expressed as $-\Delta C_q$ (*quantification cycle*) or $2^{-\Delta\Delta Cq}$ (relative quantification).

Patients with metastatic TNBC and gene expression profiling

Four women with metastatic TNBC participated to this study. For each patient, five tumor samples of a metastasis were obtained during the procedure of imagery-guided biopsy at the time of relapse, before any medical treatment. Informed written consent was obtained from the patients. The Clinical Research Board Ethics Committee (in French "Comité de Protection des Personnes") approved this study (CPP Ile-de-France N°13218)

Among the five tumor samples, i) two were formaldehyde-fixed and paraffin-embedded for histological analyses, ii) two were immediately snap-frozen in liquid nitrogen and stored in Hôpital-Saint-Louis Tumorbank for molecular analysis, iii) and one was set aside in culture medium for xenografting.

Total RNA was extracted from the frozen tumor sample as above, and transcriptomic analyses were performed using MiltenyiBiotec Microarray service. A linear T7-based amplification step was performed from 0.5 µg of all RNA samples. To produce Cy3-labeled cRNA, the RNA samples were amplified and labeled using Agilent-Quick-labeling kit. The yields of cRNA and the dye-incorporation rate were measured with ND-1000 Spectrophotometer (NanoDrop, LabTech, France). Hybridization was performed according to the Agilent 60-mer oligo-microarray processing protocol: 1.65 µg Cy3-labeled cRNA was hybridized overnight at 65°C to Agilent-Whole-Human-Genome-Oligo-Microarrays 4x44K, and fluorescence signals were detected using Agilent's Microarray-Scanner. Agilent-FE-Software determined feature intensities and quantile normalization was performed with Agi4x44PreProcess R package. Subsequent analyses were carried out with R 3.01 software (Foundation for Statistical Computing, Vienna, Austria) and based on $\log_2$ single-intensity expression data. Classification was provided by correlating gene expression profiles with the centroids for each of the 6 TNBC subtypes described by Lehmann et al [5], and with Parker centroids for PAM50 classification ([6], https:// genome.unc.edu/pubsup/breastGEO/pam50_centroids.txt.).

Patient-derived breast cancer xenografts and treatments

Four patient-derived xenografts of human TNBC were studied (XBC1 to XBC4). They had been established for a pilot study on personalized treatment for women with metastatic triple negative breast carcinoma [7], before any medical treatment of the metastatic disease.

Nude mice, purchased from Janvier (Centre-Elevage-Janvier, France), were maintained in specific pathogen-free animal housing (IUH, Paris). After imagery-guided human tumor biopsies had been performed, one sample was transported in RPMI-1640 and subcutaneously grafted in 6-week-old NMRI-nude mice, under xylasin (10mg/kg)/ketamin (100mg/kg) anaesthesia. The University Institute Board Ethics Committee for experimental animal studies approved this study (N°2012-15/728-0115).

For each xenograft model, after a successful engraftment of the metastastic sample, a clinical score was recorded daily for the mice and tumor growth was measured in two perpendicular diameters with a calliper. Tumor volumes were calculated as , L being the larger diameter (length), l the smaller (width). After mouse euthanasia, the tumor was resected, cut into small pieces of 1 mm^3, and grafted again in 20 other nude mice. The day when tumors reached a volume of 300mm3 – i.e. 100% tumor volume – was considered as Day0. Then the mice were treated over 28 days with intra-peritoneal injections of three types of chemotherapy (n=5 mice per treatment-group): epirubicin at 1 mg/kg once a week, paclitaxel at 20mg/kg twice a week, and cisplatin at 3mg/kg once a week (Supplementary Table 1 for drugs tested in TNBC xenografted mice). A daily clinical score was recorded and tumor growth measured weekly until tumor weight reached the ethically recommended limit of less than 10% of mouse weight (Directive 2010/63/EU of the European Parliament and the Council of 22 September 2010 on the protection of animals used for scientific purposes; Official Journal of the European Union L 276/33).

Ultrasonography was performed twice a week on treated and untreated mice with an AplioXT ultrasonograph (Toshiba, Japan) to assess tumor response.

Assessment of tumor response in patients

For each line of chemotherapy, the patient response under treatment was characterized. Metabolic response was assessed according to PERCIST criteria [8]. Briefly, partial metabolic response (PMR) is defined by a reduction in SUVmax of at least 30%, with no new lesions. Complete metabolic response corresponds to disappearance of all lesions in the blood-pool background. Progressive metabolic disease (PMD) is defined by an increase in SUVmax greater than 30%, or appearance of new FDG-avid lesions. Stable metabolic disease (SMD) applies when criteria for other categories (CMR, PMR or PMD) are not met.

For each line of chemotherapy, time-to-progression (TTP) was defined as the time between initiation of treatment and diagnosis of disease progression.

18-Fluoro-deoxyglucose (^{18}FDG) (5MBq/kg; not exceeding 500MBq) was injected intravenously 60 minutes before data were acquired on a Philips Gemini XL Positon Emission Tomography/Computed Tomography (PET/CT) scanner. CT data was acquired first (120kV; 100mAs; no contrast-enhancement). PET 3D data were acquired with 2 minutes per bed position, and images were reconstructed using a 3D row-action maximum likelihood algorithm (RAMLA).

PET/CT images were interpreted by a nuclear medicine physician (LV) blinded to the patient's record. ^{18}FDG uptake was expressed as standardized uptake value (SUV). A 3-dimensional region of interest (3D-ROI) was drawn around the lesions and SUV_{max} (maximum SUV value within the ROI) was measured. SUV_{max} of the lesions with the highest uptake were recorded and used for the study analysis (five target lesions were assessed). SUV_{max} of the liver was also recorded as a control value. The change in SUV_{max} at each evaluation was expressed as ΔSUV_{max} (%) = 100 × (cycle n SUV_{max} – cycle (n–1) SUV_{max})/cycle n–1 SUV_{max}. The appearance of new lesions was also recorded.

Cancer stem-cells from xenografted human TNBC: spheres and cytotoxicity

We studied spheres obtained from untreated tumor samples of XBC1 and XBC4 xenograft models. Cells expressing CD133 were obtained from dissociated sections using magnetic sorting, with anti-CD133 micro-bead antibody (Miltenyi-Biotech, Germany), using the manufacturer's procedures. We controlled CD133 cell purity by flow cytometry. CD133 positive cells were placed in a low-attachment six-well plate at a density of 2000 to 5000 cells/well, and cultured in a serum-free, high-glucose medium (DMEM-F12, Gibco, France) supplemented with 2% B27-NeuroMix and 0.4% fetal-bovine-serum (PAA, France), 5mg/mL insulin (Sigma, France), 20ng/mL epidermal-growth-factor (R&D systems, France), and 0.5ng/mL hydrocortisone (Sigma, France). Spheres were obtained after 48h of culture in a humidified chamber (37°C, 5% CO_2) under normoxia (20% O_2).

For cytotoxicity, cells from dissociated spheres were seeded in 96-well tissue culture plates at a density of 5.10^4 cells/well. After 24h of culture, they were exposed to increasing concentrations of drugs (cisplatin, epirubicin or cyclophosphamide) for 24 additional hours. Cytotoxicity was determined by the colorimetric conversion of yellow, water-soluble tetrazolium MTT (3-[4, 5-dimethylthiazol-2-yl]-2,5-diphenyl-tetrazolium-bromide; Sigma, Saint-Quentin, France), to purple, water-insoluble formazan. This reaction, catalyzed by mitochondrial dehydrogenases, is used to estimate the relative number of viable cells [9]. After incubation for 4 hours at 37°C with 0.4 mg/ml

MTT, the cells were placed in 0.1 ml of DMSO, and the absorbance was measured at 560 nm using a Fluostar Optima microplate reader (BMG LabTech, France). Experiments were performed in triplicate, untreated cells being used as positive controls, and drug-containing medium without cells as a negative control.

Experimental hypoxia, assessment of stem-cell markers and of mammosphere sensitivity to drugs

For the two xenograft models, XBC1 or XBC4, spheres were separated into two groups maintained in a humidified chamber for 48h, one under experimental hypoxia (1% O2) and the other under normoxia (20% O2). For experimental hypoxia, we chose a pO2 level of 1% as it is now accepted that stem-cell niches are hypoxic with oxygen tension as low as 1% [10].

The relative number of CD133 and CD146 expressing cells in hypoxic and normoxic spheres was assessed by flow cytometry. A total of 3.10^5 cells from hypoxic or normoxic spheres were incubated at 4°C with anti-CD133-APC (clone 293C3, Miltenyi-Biotech, Germany) and anti-CD146-PE (clone 12G5, Beckman-Coulter, France). For each antibody, corresponding isotypes were used as negative controls. Cells were analyzed on BD-Facscalibur (BD Biosciences, France) using Flow Software. Cytotoxicity tests were performed as described above.

Knock-out of autophagy gene expression in spheres

Because of a 16 day-lifetime of spheres derived from patient-derived xenografts (XBC1 and XBC4 models), CRISPR-CAS9 technology was chosen to invalidate *BECN1* and *BNIP3L* gene expression. A dedicated algorithm (see http://crispr.mit.edu website) enabled us to choose the target sequence of 20 bases around the active sites of phosphorylation for each of the two genes. Oligos designed to build the vectors containing the sgRNA1 targeting *BECN1* or *BNIP3L* genes are detailed in Supplementary Table 2. Phosphorylated active site is underlined in yellow.

5 µL of each oligo (forward and reverse) at a concentration of 10 µM was denatured for 5 minutes at 95°C, and they were then hybridized at room temperature. At this stage, hybridized oligo were cloned in a plasmid containing the Cas9 nuclease, according to manufacturer recommendations (System Bioscience Ozyme, France). 2 µg of the plasmid expressing the sgRNA and the Cas9 nuclease with 0.2 µg of a plasmid expressing GFP were added to 500 µL Opti-MEM® I Reduced Serum Medium (Life Technologies) and left for 30 minutes at room temperature. 15 µL of Lipofectamine® LTX Reagent (Life technologies) and 7.5 µL de PLUS™ Reagent were added to the medium, which was deposited in a 6-well plate together with 2.10^5 cells from spheres derived from our tumor xenografts, and incubated for 4 hours at 37°C. After incubation for 24 hours, 1% Kanamycin (G418, Life Technologies) was used to select cells that had integrated the plasmid.

Droplet digital PCR

Droplet digital PCR was performed to assess the copy number of *BECN1* or *BNIP3L* genes in spheres transfected with empty plasmid and spheres transfected with sg1*BECN1* or sg1*BNIP3L*. 48h after transfection, and after antibiotic selection, spheres were processed for RNA extraction and reverse transcription.

Each droplet digital PCR assay, performed according to the MIQE guidelines (minimum information for publication of quantitative real-time PCR experiment) [11], was conducted in triplicate. Reagent mixes were prepared using standard Taqman primer/probe chemistry with a 2XddPCR Mastermix (BioRad, Laboratories, CA), a 20X primer/probe (900/250 nM), and 5 µL sample cDNA template in a final volume of 20 µL. The reagent mixture was loaded into an eight-channel droplet generator (BioRad, Laboratories, CA). 60 µL of droplet generation oil was loaded for each channel and after generation of water-in-oil droplets the droplets were transferred to a 96-well PCR plate and placed in a Biorad thermocycler. An initial denaturation step (95°C, 10 min) was followed by 45 cycles at 95°C for 15 sec and at 60°C for 1 min. The PCR products were streamed through a droplet reader and the results analysed using QuantaSoft software (BioRad Laboratories, CA). All droplets were gated on the basis of detector peak width to exclude doublets or triplets.

Electron microscopy of spheres KO for BECN1 or BNIP3L

Spheres transfected with empty plasmid and spheres transfected with sg1*BECN1* or sg1*BNIP3L* were dissociated and cells in suspension were counted. After centrifugation, a pellet of 10^6 cells was further processed for electron microscopy, and fixed in 2% glutaraldehyde-buffered 0.1M cacodylate and embedded in epoxy resin. Ultrathin sections were stained with uranyl acetate and lead citrate.

Ultrastructural analysis, performed on a Hitachi-7650, focused on the cytoplasms of tumor cells to detect characteristic autophagosomes according to the "Guidelines for the use and interpretation of assays for monitoring autophagy" [12].

A quantitative study was performed comparing the numbers of autophagosomes per cytoplasmic area in cells transfected with empty plasmid versus sg1*BECN1* or sg1*BNIP3L* transfected cells (Supplementary Figure 2). We used CellSens Dimension 1.9 software (Olympus) to delineate and calculate the cytoplasmic areas of tumor cells on ultrastructural images at magnification 20.000. The cytoplasmic area was calculated in 25 tumor cells in each series of spheres. Within these cytoplasmic areas, the numbers of autophagosomes were counted, and the results

expressed as the mean numbers of autophagosomes per 1000 µm² of cytoplasmic area.

Statistical analyses

The statistical analyses were performed using R 2.15.2 statistical software (R-Foundation for Statistical Computing, Vienna/Austria). The differences between the two groups of patients (pCR and non-pCR), for the pre-treatment numbers of CD133-expressing cells, ADLH1-expressing cells, ALDH1/CD133-coexpressing cells, and the presence of clustered cells or necrosis, were assessed using Wilcoxon signed-rank tests with the wilcox. test R command. Probabilities were corrected for multiple comparisons using the Bonferroni method with the p.adjust R command. A p value ≤ 0.05 was considered to be significant. Principal component analysis and correlation circle were performed with the ade4 R-package.

Among non-pCR patients, the differences between tumor cells and cancer stem-cells, for the number of Ki67-expressing cells, TUNEL-expressing cells, BECLIN1-, BNIP3L-, or LC3B-expressing cells, were assessed using the same test and the same correction. Variables significantly predicting pCR were also selected by multivariate regression with the glm command (binomial model) in the R stats package and by forward selection with the stepAIC algorithm in the R MASS package. Association between pCR and relapse was assessed by Cox regression with the R survival package.

For cell counts in immuno-stained tissue sections, flow cytometry, and MTT results, and for RT-qPCR results, the mean ±SEM (standard error mean) was calculated in each experimental group and shown in bar graphs. Quantitative values were compared using Student's *t*-test (two-tailed). P values under 0.05 (after Bonferroni correction for multiple comparisons) were considered significant. Comparisons between tumor growth curves were analysed globally with permutation tests with the R statmod package and two-by-two comparisons were carried out for critical points. For correlation studies, the Kendall rank correlation coefficient was calculated between patient ΔSUVmax for one chemotherapy regimen and the coefficient of inhibition for the same regimen in TNBC xenograft.

REFERENCES

1. Gavrieli Y, Sherman Y, Ben-Sasson SA. Identification of programmed cell death in situ via specific labeling of nuclear DNA fragmentation. J Cell Biol. 1992; 119:493–501.
2. Schlafli AM, Berezowska S, Adams O, Langer R, Tschan MP. Reliable LC3 and p62 autophagy marker detection in formalin fixed paraffin embedded human tissue by immunohistochemistry. Eur J Histochem. 2015; 59:2481.
3. Rademakers SE, Lok J, van der Kogel AJ, Bussink J, Kaanders JH. Metabolic markers in relation to hypoxia; staining patterns and colocalization of pimonidazole, HIF-1alpha, CAIX, LDH-5, GLUT-1, MCT1 and MCT4. BMC Cancer. 2011; 11:167.
4. Yang MH, Wu MZ, Chiou SH, Chen PM, Chang SY, Liu CJ, Teng SC, Wu KJ. Direct regulation of TWIST by HIF-1alpha promotes metastasis. Nature cell biology. 2008; 10:295–305.
5. Lehmann BD, Bauer JA, Chen X, Sanders ME, Chakravarthy AB, Shyr Y, Pietenpol JA. Identification of human triple-negative breast cancer subtypes and preclinical models for selection of targeted therapies. The Journal of clinical investigation. 2011; 121:2750–2767.
6. Parker JS, Mullins M, Cheang MC, Leung S, Voduc D, Vickery T, Davies S, Fauron C, He X, Hu Z, Quackenbush JF, Stijleman IJ, Palazzo J, et al. Supervised risk predictor of breast cancer based on intrinsic subtypes. J Clin Oncol. 2009; 27:1160–1167.
7. Bousquet G, Feugeas JP, Ferreira I, Vercellino L, Jourdan N, Bertheau P, de Bazelaire C, Barranger E, Janin A. Individual xenograft as a personalized therapeutic resort for women with metastatic triple-negative breast carcinoma. Breast cancer research. 2014; 16:401.
8. Wahl RL, Jacene H, Kasamon Y, Lodge MA. From RECIST to PERCIST: Evolving Considerations for PET response criteria in solid tumors. Journal of nuclear medicine. 2009; 50 Suppl 1:122S-150S.
9. Mosmann T. Rapid colorimetric assay for cellular growth and survival: application to proliferation and cytotoxicity assays. Journal of immunological methods. 1983; 65:55–63.
10. Mohyeldin A, Garzon-Muvdi T, Quinones-Hinojosa A. Oxygen in stem cell biology: a critical component of the stem cell niche. Cell Stem Cell. 2010; 7:150–161.
11. Bustin SA, Benes V, Garson JA, Hellemans J, Huggett J, Kubista M, Mueller R, Nolan T, Pfaffl MW, Shipley GL, Vandesompele J, Wittwer CT. The MIQE guidelines: minimum information for publication of quantitative real-time PCR experiments. Clinical chemistry. 2009; 55:611–622.
12. Klionsky DJ, Abdalla FC, Abeliovich H, Abraham RT, Acevedo-Arozena A, Adeli K, Agholme L, Agnello M, Agostinis P, Aguirre-Ghiso JA, Ahn HJ, Ait-Mohamed O, Ait-Si-Ali S, et al. Guidelines for the use and interpretation of assays for monitoring autophagy. Autophagy. 2012; 8:445–544.

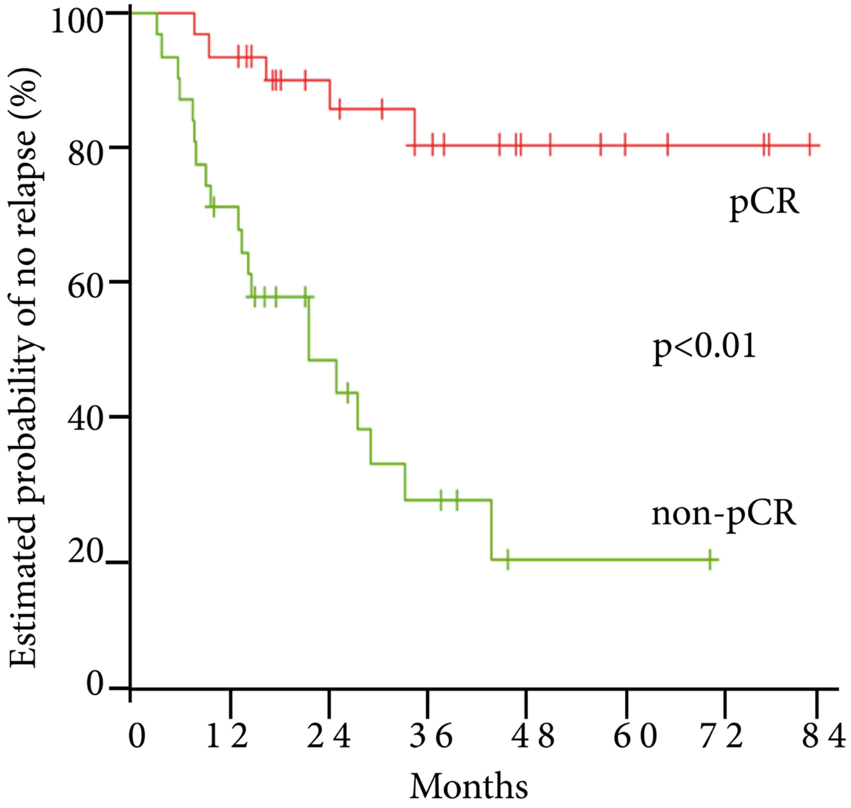

Supplementary Figure 1: Probability of relapse over time according to pCR for women receiving neoadjuvant chemotherapy for the treatment of localized TNBC. The probability of relapse over time is significantly higher for women in the pCR group than for women in the non-pCR group after neoadjuvant chemotherapy. pCR: pathological complete response.

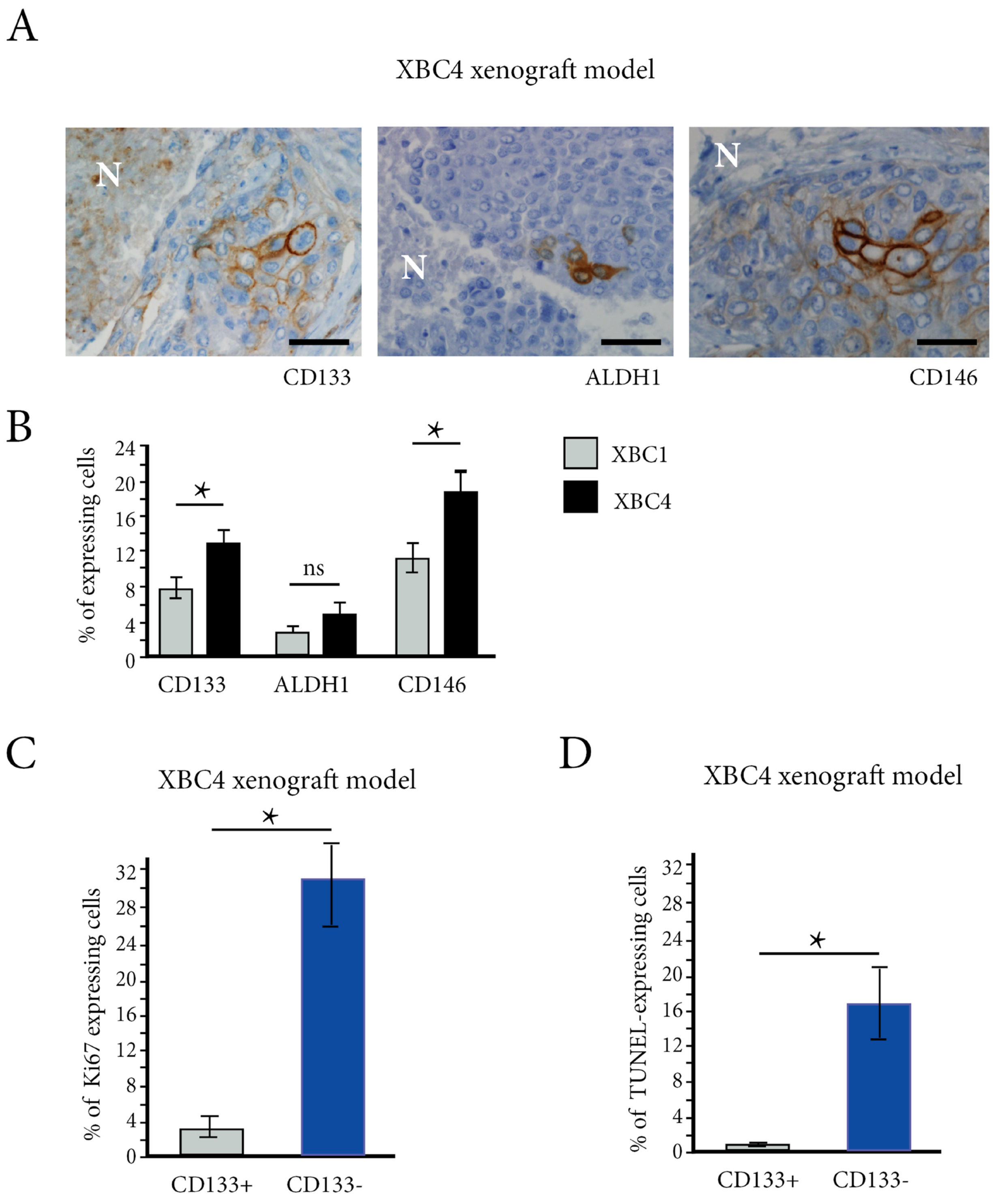

Supplementary Figure 2: Patient-derived TNBC xenografts reproduce breast cancer stem-cell characteristics observed in patient pre-treatment biopsies. (**A**) CD133, ALDH1, and CD146 expressing cells are distributed in peri-necrotic areas (N) in XBC4 chemoresistant model. Indirect immunoperoxydase method. ×400 magnification. (**B**) CD133, and CD146 expressing cells are significantly more numerous in XBC4 than XBC1 model. *: p <0.05. (**C**) In XBC4 xenograft, CD133-expressing cells do not co-express Ki67, nor TUNEL, while CD133-negative cells do. *: p <0.05.

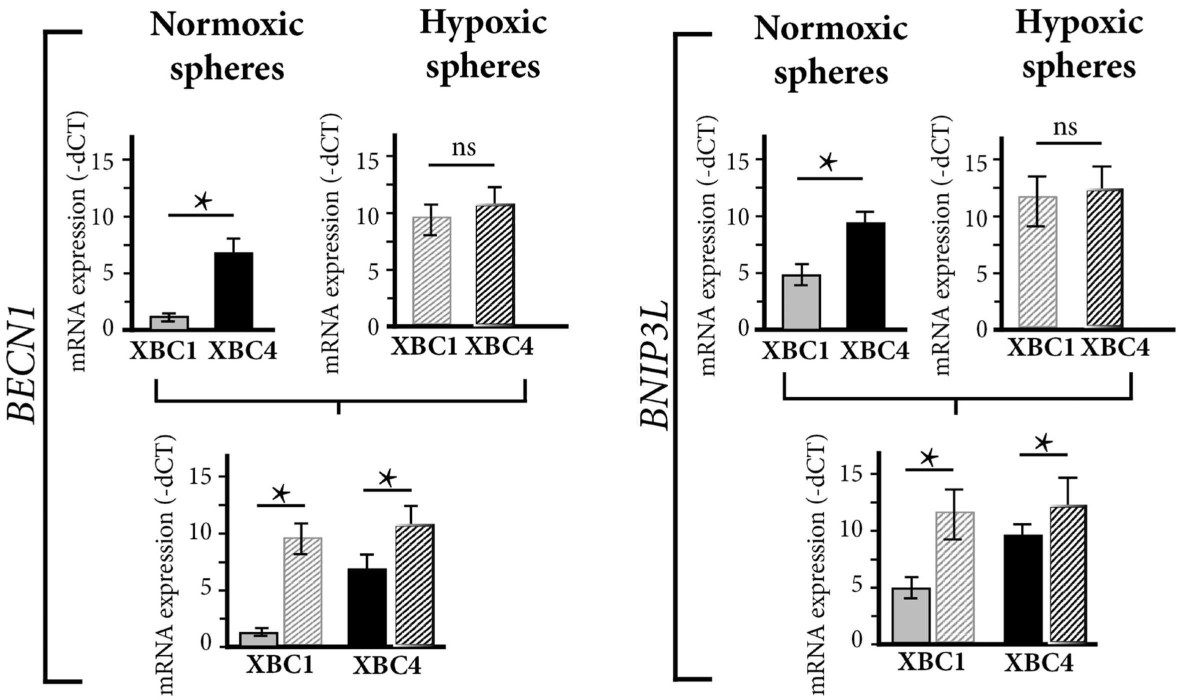

Supplementary Figure 3: Autophagic molecular markers in XBC1 and XBC4 spheres. Under normoxic conditions, mRNA level expression of *BECN1* or *BNIP3L* is significantly higher in spheres derived from XBC4 model than in spheres derived from XBC1 model. Experimental hypoxia significantly increases *BECN1* and *BNIP3L* mRNA levels in both models. *:$p < 0.05$, ns:not significant.

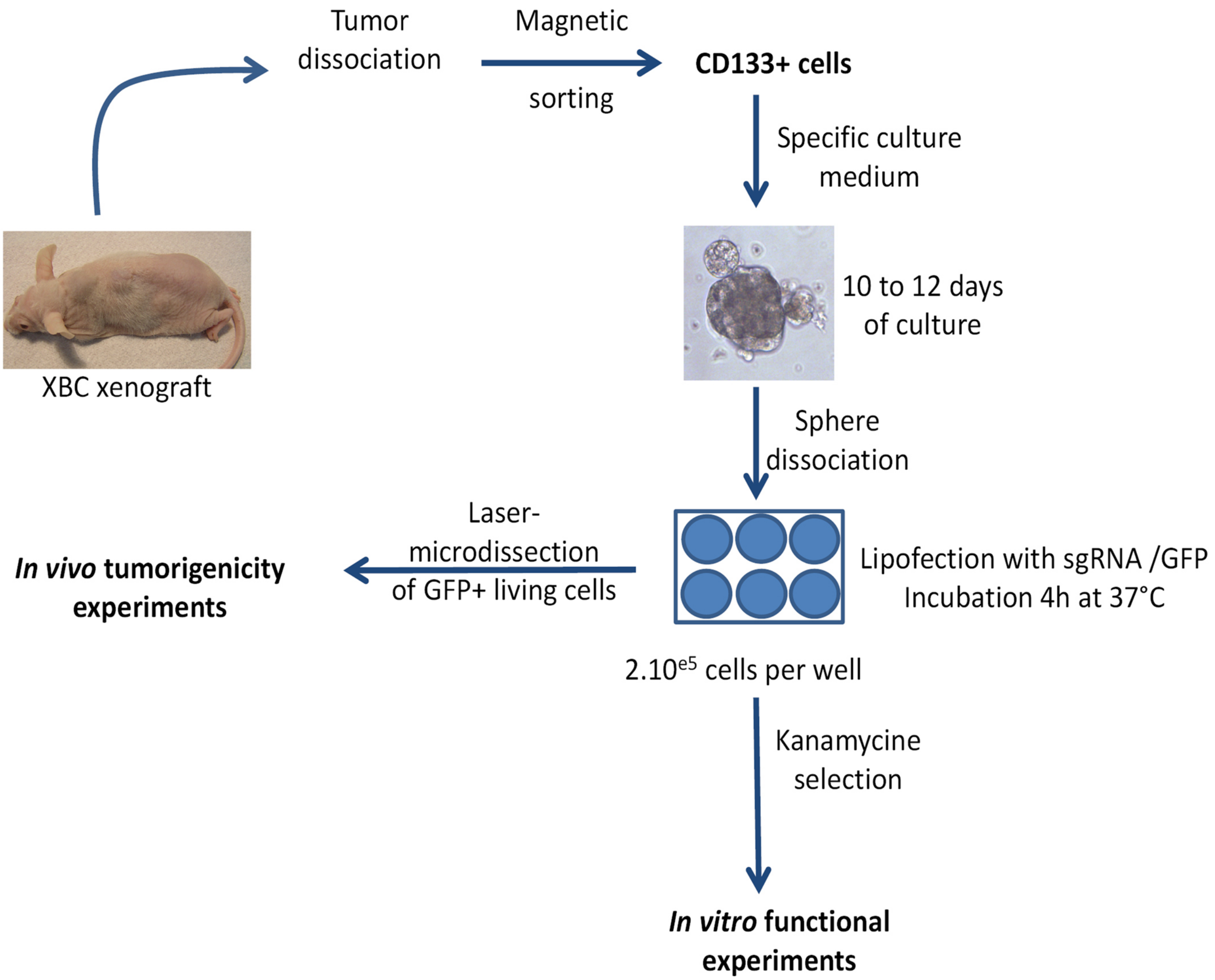

Supplementary Figure 4: spheres derived from XBC models and related experiments: a diagram. The diagram schematizes the different steps required to obtain mammospheres in culture from a tumor xenograft, and their further use for *in vitro* functional experiments and *in vivo* tumorigenicity experiments.

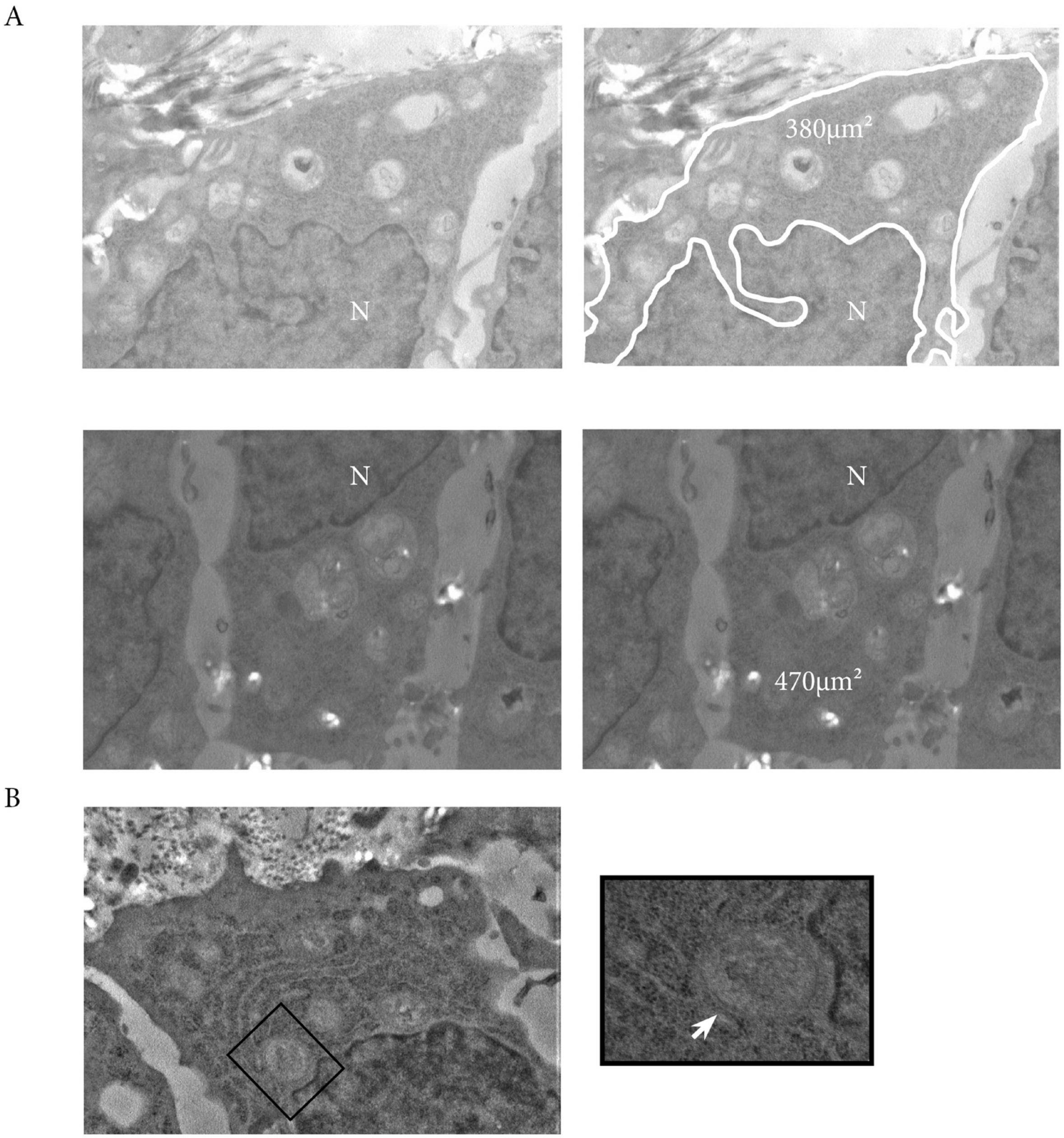

Supplementary Figure 5: Electron microscopy of XBC4 non-transfected spheres. (**A**) At X20.000 magnification, cytoplasmic areas of tumor cells are delineated (white line) and calculated with a dedicated software, to exclude the nucleus area (N). (**B**) In these cytoplasmic areas, autophagosomes with characteristic double-membrane (white arrow at higher magnification X70.000) are counted.

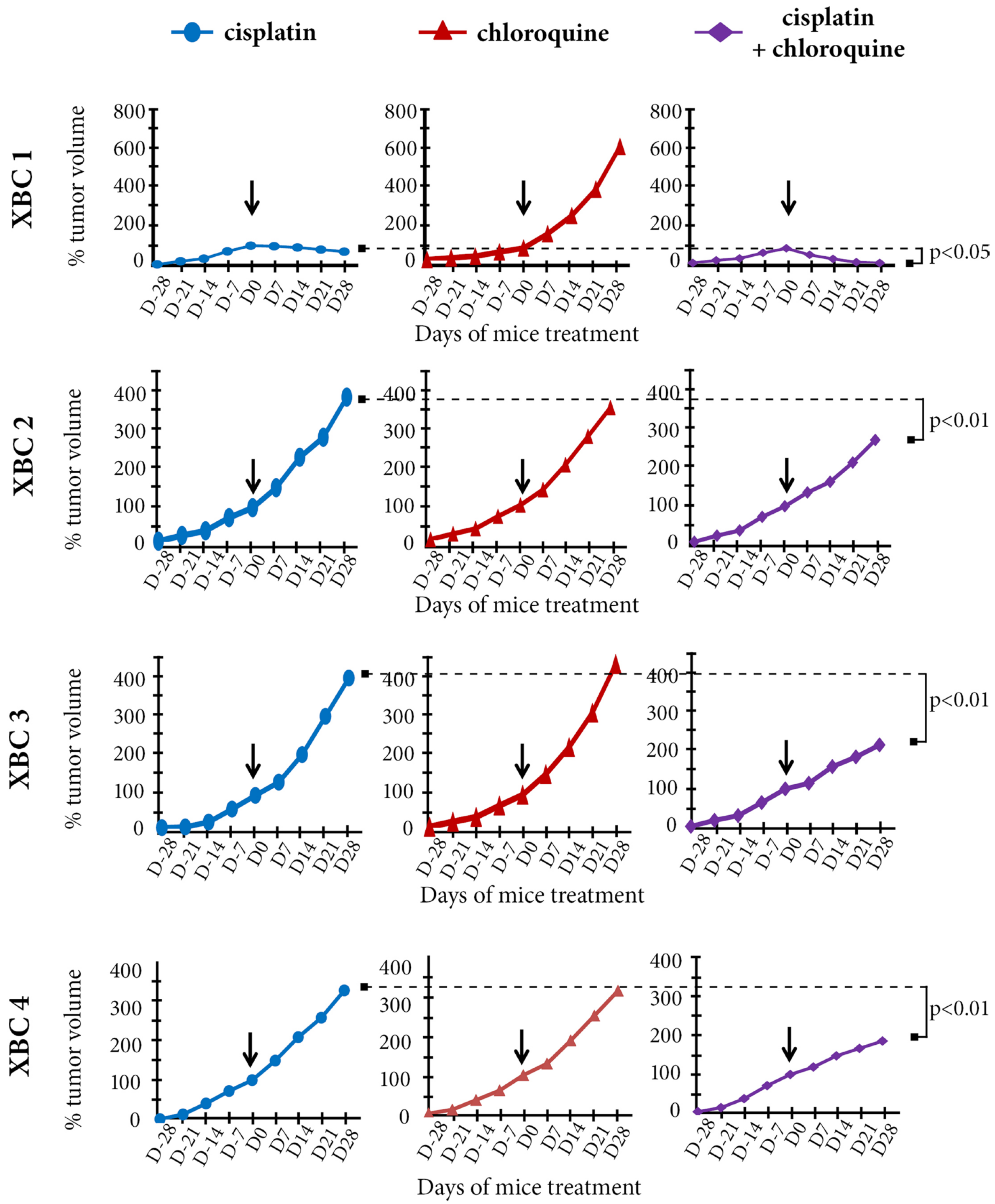

Supplementary Figure 6: Treatment of XBC2, XBC3, and XBC4 cisplatin-resistant models with chloroquine does not induce a significant tumor growth inhibition at Day 28. Chloroquine added to cisplatin significantly reduces tumor growth at Day28, when compared to cisplatin alone ($p < 0.01$) and to chloroquine alone ($p < 0.01$).

Supplementary Table 1: Mean percentages of proliferation, apoptosis, and autophagy before treatment in the 58 non-pCR patients

	CD133-negative cells		CD133-positive cells		*p*
	%	±SD	%	±SD	
Ki67	29	25.2	4.5	2.6	< 0.001
TUNEL	20.5	11.5	0.5	0.1	< 0.05
BECLIN1	5.8	3.2	68	26.5	< 0.001
LC3B	22	3.9	79	19.5	< 0.05
BNIP3L	18	9.8	75	29.2	< 0.05

Supplementary Table 2: Patients' characteristics at the time of the transcriptomic analysis and of patient-derived xenografts in mice

Patients	Age (yr)	TNM	Metastases	Site of biopsy	Classification	
					PAM50	(Lehmann et al., 2011)
Patient 1	30	T4cN2M1	Lymph nodes, lung	Breast	Basal	BL1
Patient 2	45	T2N2M1	Lymph nodes	Breast	Basal	BL2
Patient 3	42	T4dN1M1	Lymph nodes	Breast	Basal	BL2
Patient 4	66	T1N0M1	Lung, liver, bone, lymph nodes	Lymph node	Basal	Stm

TNM refers to international Tumor-Node-Metastasis classification.

PAM50: gene set of 50 genes used to classify breast cancer sub-types.

BL1: basal-like 1; BL2: basal-like 2; Stm: stem-like.

Lehmann BD, Bauer JA, Chen X, Sanders ME, Chakravarthy AB, Shyr Y, Pietenpol JA. . Identification of human triple-negative breast cancer subtypes and preclinical models for selection of targeted therapies. The Journal of clinical investigation. 2011; 121:2750–2767.

Supplementary Table 3: Response to treatment in patients and corresponding patient-derived xenografts

Patients	Treatment	Best response in patients/ΔSUVmax	Coefficient of inhibition in corresponding PDX
Patient 1	SIM	PMR/-0.56	–1.61
	Docetaxel	PMD/0.42	1.18
	Cisplatin Gemcitabine	SMD/0.03	–1.42
Patient 2	Cisplatin Gemcitabine	PMD/2.1	1.52
	Capecitabine Bevacizumab	SMD/0.02	–0.88
	Paclitaxel Bevacizumab	PMR/-0.88	–1.67
Patient 3	SIM	SMD/0.07	0.61
	Docetaxel	PMD/2.18	1.67
Patient 4	Paclitaxel Bevacizumab	PMD/0.31	1.57
	Capecitabine Bevacizumab	PMD/0.41	1.2
	Sunitinib	PMD/2.13	3.59
	Cisplatin Everolimus	PMD/0.47	0.98

SUV: standardized uptake value.
PDX: patient-derived xenograft.
SIM = dose-dense epirubicin-cyclophosphamide regimen [{Lehmann-Che, 2010 #13}]
PMR = partial metabolic response, PMD = progressive metabolic disease, SMD = stable metabolic disease.

Supplementary Table 4: IC50 (µmol/L) for spheres derived from TNBC xenografts, under normoxic or hypoxic conditions

	XBC1		XBC4	
Cisplatin				
normoxia	19 ± 1.6	$p < 0.05$	70 ± 5.0	ns
hypoxia	40 ± 2.6		79 ± 9.6	
Epirubicin				
normoxia	0.12 ± 0.03	$p < 0.05$	0.21 ± 0.01	ns
hypoxia	0.25 ± 0.07		0,27 ± 0,05	
Paclitaxel				
normoxia	0.22 ± 0.04	$p < 0.05$	1.84 ± 0.14	ns
hypoxia	0.75 ± 0.07		2.10 ± 0.80	

ns : not significant.

Supplementary Table 5: IC50 (µmol/L) for XBC4 spheres, transfected with empty plasmids, or transfected with sg1*BECN1* or with sg1*BNIP3L*

	XBC4 spheres transfected with					
	empty plasmid	sg1*BECN1*		empty plasmid	sg1*BNIP3L*	
Cisplatin	70 ± 5.0	39 ± 0.35	$p < 0.05$	70 ± 5.0	40 ± 0.39	$p < 0.05$
Epirubicin	0.21 ± 0.01	0.11 ± 0.01	$p < 0.05$	0.2 ± 0.01	0.13± 0.02	$p < 0.05$
Paclitaxel	1.84 ± 0.14	0.62 ± 0.09	$p < 0.05$	1.8 ± 0.14	1.2 ± 0.24	$p < 0.05$

Supplementary Table 6: Drugs tested in TNBC xenografted mice

Drug	Dose	Mode of administration	Frequency of administration
Epirubicin	1 mg/kg	Intra-peritoneal	Once a week
Paclitaxel	20 mg/kg	Intra-peritoneal	Twice a week
Cisplatin	3 mg/kg	Intra-peritoneal	Once a week

Supplementary Table 7: Oligo sequences designed to build the vectors containing the sgRNA1 targeting *BECN1* or *BNIP3L* genes

Targeted gene	Forward sequence	Reverse sequence
BECN1	BECLFwguide1bottomstrandsgRNA<br>5′TGTATGAGACCACGCCCC AGTGACCTGGAAGTGT3′	BECLRvguide1bottomstrandsgRNA<br>5′AAACACACTTCCAGGTCA CTGGGGCGTGGTCTCA 3′
BNIP3L	bnipFwguide7bottomstrandsgRN<br>5′TGTATGAGACCACGCTTCT TCTGACTGAGAGCTA3′	bnipRvguide7bottomstrandsgRNA<br>5′AAACTAGCTCTCAGTCAGA AGAAGCGTGGTCTCA3′

Phosphorylated active site is underlined in yellow.

Supplementary Table 8: Treatment with chloroquine to reverse cisplatin-resistance in TNBC xenografted mice

Drug	Dose	Mode of administration	Frequency of administration
Cisplatin	3 mg/kg	Intra-peritoneal	Once a week
Chloroquine	50 mg/kg	Intra-peritoneal	Every day
Cisplatine<br>+<br>Chloroquine	3 mg/kg<br><br>50 mg/kg	Intra-peritoneal	Once a week<br><br>Every day
